# Precise Identification of Recurrent Somatic Mutations in Oral Cancer Through Whole-Exome Sequencing Using Multiple Mutation Calling Pipelines

**DOI:** 10.3389/fonc.2021.741626

**Published:** 2021-11-29

**Authors:** Li-Han Lin, Chung-Hsien Chou, Hui-Wen Cheng, Kuo-Wei Chang, Chung-Ji Liu

**Affiliations:** ^1^ Department of Medical Research, MacKay Memorial Hospital, Taipei, Taiwan; ^2^ Institute of Oral Biology, School of Dentistry, National Yang Ming Chiao Tung University, Taipei, Taiwan; ^3^ Department of Stomatology, Taipei Veterans General Hospital, Taipei, Taiwan; ^4^ Department of Oral and Maxillofacial Surgery, Taipei MacKay Memorial Hospital, Taipei, Taiwan

**Keywords:** mutation burden, oral cancer, somatic mutation, survival, whole-exome sequencing

## Abstract

Understanding the genomic alterations in oral carcinogenesis remains crucial for the appropriate diagnosis and treatment of oral squamous cell carcinoma (OSCC). To unveil the mutational spectrum, in this study, we conducted whole-exome sequencing (WES), using six mutation calling pipelines and multiple filtering criteria applied to 50 paired OSCC samples. The tumor mutation burden extracted from the data set of somatic variations was significantly associated with age, tumor staging, and survival. Several genes (*MUC16*, *MUC19*, *KMT2D*, *TTN*, *HERC2*) with a high frequency of false positive mutations were identified. Moreover, known (*TP53*, *FAT1*, *EPHA2*, *NOTCH1*, *CASP8*, and *PIK3CA*) and novel (*HYDIN*, *ALPK3*, *ASXL1*, *USP9X*, *SKOR2*, *CPLANE1*, *STARD9*, and *NSD2*) genes have been found to be significantly and frequently mutated in OSCC. Further analysis of gene alteration status with clinical parameters revealed that canonical pathways, including clathrin-mediated endocytotic signaling, NFκB signaling, PEDF signaling, and calcium signaling were associated with OSCC prognosis. Defining a catalog of targetable genomic alterations showed that 58% of the tumors carried at least one aberrant event that may potentially be targeted by approved therapeutic agents. We found molecular OSCC subgroups which were correlated with etiology and prognosis while defining the landscape of major altered events in the coding regions of OSCC genomes. These findings provide information that will be helpful in the design of clinical trials on targeted therapies and in the stratification of patients with OSCC according to therapeutic efficacy.

## Introduction

Oral squamous cell carcinoma (OSCC) is one of the most common malignancies of the upper aerodigestive tract, with poor prognosis and high mortality rates. In 2020 alone, 377,713 new cases of OSCC were diagnosed worldwide, among whom 177,757 have died from their disease (1). OSCC generally develops as a result of multi-step carcinogenic processes (2). Meanwhile, approximately 4%–7.4% of the patients have been found to develop simultaneous tumors which are located in the head and neck region (3, 4). Moreover, multiple lesions may develop concurrently and over large mucosal areas, subsequently progressing into cancers. This may be the reason for the high recurrence of OSCC after treatment (5), as well as the increased incidence and mortality of OSCC worldwide (6). Therefore, understanding the genomic alterations which are associated with OSCC carcinogenesis is crucial for appropriate diagnosis and therapy.

Recent developments in high-throughput next-generation parallel sequencing technologies have facilitated the sensitive detection and quantification of genetic alterations in tumor biopsies. In line with this, whole-exome sequencing (WES) has provided new insights into the molecular basis of head and neck squamous cell carcinoma (HNSCC) progression (7, 8). WES data obtained from the Cancer Genome Atlas (TCGA) (9, 10) has further highlighted this molecular complexity by identifying novel significantly mutated genes (11). Therefore, to improve the diagnosis of individuals at risk and the treatment of patients, more sensitive and specific biomarkers for OSCC need to be established (10, 12). Previous exome sequencing studies on HNSCC have consistently revealed that *TP53*, *CDKN2A*, *PIK3CA*, *HRAS*, and *NOTCH1* were significantly mutated (7, 8). Another genomic analysis of OSCC in Taiwan revealed that the *CHUK* and *ELAVL1* genes were significantly and frequently mutated (13). Moreover, frequently and recurrently mutated genes, including *USP9X*, *MLL4*, *ARID2*, *UNC13C*, and *TRPM3*, have been reported in gingivo-buccal oral squamous cell carcinomas (14). The accumulation of somatic mutations within a cancer genome has revealed that certain oncogenic patterns are associated with the exposure to mutagens and with defects in DNA repair (15, 16).

Several packages for the analysis of genomic data with different algorithms have been applied to increase the accuracy of mutation detection. Nonetheless, significant discrepancies between the results of different algorithms have been observed, leading to difficulties in selecting candidate mutations for validation (17, 18). To date, no single study has been able to exhaustively address all possible relevant issues in variant calling. Hence, the current study sought to contribute towards addressing this concern by comparing six variant callers using data from 50 matched-paired OSCC samples which were sequenced on a whole-exome platform. Circularity in defining false-negative mutations was minimized by relying on previous high-quality work using independent data and variant calling methods. Selected data were then combined and validated using Sanger sequencing and Integrative Genomics Viewer (IGV) to greatly reduce false-positive calls while maintaining sensitivity for detecting genuine mutations. Thereafter, truly somatic mutations were analyzed using clinical data. 

## Methods

### Participants and Data Collection

50 patients with OSCC were enrolled in this study after providing informed consent. This study was approved by the institutional review board of MacKay Memorial Hospital (approval numbers: 12MMHIS178 and 15MMHIS104). Tumor specimens were collected from patients during OSCC surgery. Laser capture microdissection was performed to isolate relatively pure tumor cells for DNA extraction according to previously established protocols (19). 10 mL of whole blood were collected in Vacutainer tubes containing ethylenediaminetetraacetic acid as anticoagulant (Becton Dickinson, Franklin Lakes, NJ). Genomic DNA was extracted from blood or tumor specimens using the QIAamp DNA Blood Mini Kit according to the manufacturer’s instructions (QIAGEN, Hilden, Germany).

Demographic data, including age, sex, clinical stage, perineural invasion, and lymphovascular invasion were retrospectively obtained from the patients’ medical records. Clinical staging was performed according to the American Joint Committee on Cancer (AJCC 7th edition) guidelines for tumor, node, and metastasis TNM classification (20). None of the patients which were enrolled in this study had received adjuvant chemotherapy or radiotherapy before surgery. WES data from the TCGA-HNSCC dataset was collected and downloaded from the Genomic Data Commons portal (https://portal.gdc.cancer.gov/). Using the TCGA-HNSCC dataset, all cases where the primary site was located at the tongue, lip, mouth floor, tonsil, gums, palate, or oropharynx, were included. This dataset was called “TCGA-OSCC” dataset. In total, 387 OSCC patients with somatic mutation data were analyzed herein. Only mutations in the coding regions and in splicing sites were retained, whereas mutations in the introns, intergenic regions, and in untranslated regions (UTR) were filtered out.

### Whole-Exome Sequencing

WES was performed with the SureSelect Human All Exon v6 + UTR Enrichment Kit (Agilent, Santa Clara, CA), followed by sequencing on a NextSeq500 DNA sequencer (Illumina, San Diego, CA). Downstream analysis was performed as previously described (21). The software used for WES analysis is listed in **Table S1**. Somatic mutation was called using our pipelines were shown in **Figure S1**. Sequencing data was aligned to the human genome (NCBI build GRCh38/UCSC hg38) using BWA-MEM. SAMtools was used for the file format conversion from SAM to BAM. Six different programs were used to call somatic mutations, including two callers (Muse and SomaticSniper) for single nucleotide variants (SNVs) (22, 23) and four callers (Mutect2, Strelka2, VarScan2, and VarDict) for both SNVs and short insertion and deletion variants (indels) (9, 23–26). These variant callers were run with default parameters and further filtering of the data was based on the following criteria: (1) Mutations called by Muse, SomaticSniper, and Mutect2 were labeled as “PASS” in the FILTER column, (2) Strelka2 algorithm with WES default parameters was used to identify somatic mutations (27). Only variants labeled “PASS” were considered high-quality variants (28). (3) Mutations called by VarScan2 were labeled as “Somatic” in the somatic status column, and (4) mutations called by VarDict were labeled as “StrongSomatic” in the INFO column. Thereafter, mutations outside the targeted region were removed. The filtered mutations were considered somatic mutations. The command line calls somatic mutations as described in **Table S2**. All WES data in this manuscript were submitted to Short Reads Archive under the BioProject accession PRJNA749133 and SRA Run Selector project (https://www.ncbi.nlm.nih.gov/Traces/study/?acc=PRJNA749133&o=acc_s%3Aa).

The somatic mutations were annotated using Ensembl Variant Effect Predictor (version 102, https://asia.ensembl.org/Homo_sapiens/Tools/VEP) (21). Potential mutational driver genes in OSCC were identified and annotated using the InToGene platform (https://www.intogen.org/search) and Bailey et al. datasets (29, 30).

### Filtering Strategies

To reduce false-positive somatic mutations which might originate from germline mutations or might have been accidentally generated during sample preparation, DNA amplification, sequencing, and ambiguous mapping (31), the following filter flags were used to annotate and filter somatic mutations: (1) removing common polymorphisms (SNPs): minor allele frequency in the 1000 Genomes Project or The Genome Aggregation Database (gnomAD) > 1%; (2) removing Panel-of-Normal (PoN): A normal panel was created from 50 normal samples using GATK (Genome Analysis Toolkit) tool CreateSomaticPanelOfNormals; (3) removing oxodG artifacts: oxidation-damaged base 8-oxodG (8-oxoguanine) was identified and filtered using GATK tools CollectSequencingArtifactMetrics and FilterByOrientationBias; (4) removing strand bias, multiallelic site, and clustered events: strand bias is a type of sequencing bias wherein one DNA strand is favored over another by the variant. A multiallelic site is a genomic locus that contains two or more alternative (Alt) alleles. Clustered events are several variants that are clustered in a region of the genome. These artifacts were estimated and filtered using the GATK tools CalculateContamination, GetPileupSummaries, and FilterMutectCalls; and (v) Alt allele count filter: we filtered out mutations with Alt alleles in tumor derived date (T_Alt) < 4 and mutations with Alt alleles in normal data (N_Alt) ≥ 4 (**Figure 2A**) (32).

### Merging Results From Multiple Callers

Somatic mutations were called using multiple callers and stored as VCF (Variant Call Format) files. Following variant calling and filtering, VCF files from six tools were merged according to each sample ID and genomic position (e.g., ID-chr1-123). The number of variants hits detected for each mutation in the merge files was then counted. Thereafter, mutations that were not identified by two or more variant callers were removed. The tumor mutation burden (TMB) was calculated using the number of non-synonymous mutations per mega-base (Mb) in the target region of SureSelect Human All Exon v6 + UTR (91.08 Megabases). The target region BED file is available online at the SureDesign website (https://earray.chem.agilent.com/suredesign/).

### Mutation Validation

Sanger sequencing and IGV was performed to validate somatic mutations (33, 34). For Sanger sequencing, individual primer sets designed by Primer3 (version 0.4.0) are listed in **Table S3**. Polymerase chain reactions (PCRs) were performed using the KAPA LongRange HotStart PCR Kit (KAPA Biosystems, Wilmington, MA, USA). Amplicons were sequenced on an ABI 3730xl DNA Analyzer (Applied Biosystems, Foster City, CA, USA) with the BigDye Terminator Cycle Sequencing Kit (Applied Biosystems).

The top 20 most frequently mutated genes [mutated in at least 7/50 (14%) patients] were selected and examined for false-positive rates using IGV. Mutations were considered “true-positive” based on the following criteria: (1) number of Alt alleles < 3 in normal cells and ≥ 3 in tumors; (2) both forward and reverse strands have at least one mutant allele; (3) number of mismatches within a 40 bp window ≤3; and (4) allelic configurations of the mutation are multiallelic variants (21).

### Visualization of WES Data

The 200 most frequently mutated genes [mutated in at least 4/50 (8%) patients] in our data and in the TCGA-OSCC dataset were selected for the creation of a circular plot using Circos-0.69 (http://circos.ca/software/). Track 1 (inner circle) visualizes the mutation frequency of the genes in the TCGA-OSCC dataset (**Figure 4**), whereas Track 2 (outer cycle) illustrates the genomic profile from our WES data.

### Pathway Analysis

The 200 most frequently mutated genes in our study were imported into the analysis pipeline of the Ingenuity Pathway Analysis (IPA, QIAGEN, CA, USA; http://www.ingenuity.com/products/pathways_analysis.html). IPA was used to examine which canonical pathways were enriched within our candidate genes. In IPA, Fisher’s exact test was used to determine whether a canonical pathway was enriched within a data set. A −log [*P* value] > 1.3 (corresponding to a *P* value of <0.05) was set as the threshold for statistical significance. The United States Food and Drug Administration (FDA) Table of Pharmacogenomic Biomarkers in Drug Labels was used to match our candidate genes against FDA-approved drugs (https://www.fda.gov/). 

### Statistical Analysis

Data are presented as mean ± standard error of mean. The Chi-squared test, Fisher’s exact test, and Mann–Whitney U test were used for statistical analysis. The receiver operating characteristic curve (ROC) analysis was used to identify the optimal thresholds of read-depth (DP) and minor allele frequency (MAF) in mutation calling. Overall survival (OS) was defined as the duration from the first date of diagnosis to death or last date of follow-up. Kaplan–Meier analysis was used to compare the OS between two groups. *P* values less than 0.05 were considered to be statistically significant.

## Results

### Patient Characteristics

In this study, we have collected and analyzed tumor specimens and matched blood samples from 50 patients with OSCC. 48 patients were male and 2 were female, with an average age of 59.6 years (range: 40–89 years). All patients were confirmed to have squamous cell carcinoma. The most common primary sites were the bucca (28%, 14/50) and the gingiva (24%, 12/50). The detailed clinical characteristics of our study subjects are described in **Table 1**.

**Table 1:** Association between tumor burden and clinical parameters.

Parameter	N	mean ± SEM	*P*-value
Age			
≤ 60	23	1.403 ± 0.383	0.034*
> 60	27	2.312 ± 0.664	
Gender			
Male	48	1.94 ± 0.416	0.488
Female	2	0.786 ± 0.006	
T stage			
T1-3	13	1.351 ± 0.442	0.177
T4	37	2.085 ± 0.518	
N stage			
N0	28	2.282 ± 0.678	0.464
N+	22	1.400 ± 0.283	
Clinical stage			
I-III	11	0.801 ± 0.169	0.021*
IV	39	2.202 ± 0.502	
Differentiation			
Well	36	2.166 ± 0.545	0.538
Moderate-poor	14	1.194 ± 0.235	
Perineural invasion			
No	33	1.182 ± 0.334	0.301
Yes	17	2.039 ± 1.006	
Lymphovascular invasion			
No	39	1.885 ± 0.471	0.399
Yes	11	1.926 ± 0.764	
HPV status			
p16 negative	46	1.989 ± 0.433	0.453
p16 positive	4	0.804 ± 0.173	

Statistical test for comparing two groups by Mann Whitney U-test.

*P < 0.05.

### Variant Calling in OSCC

**Figure 1:**
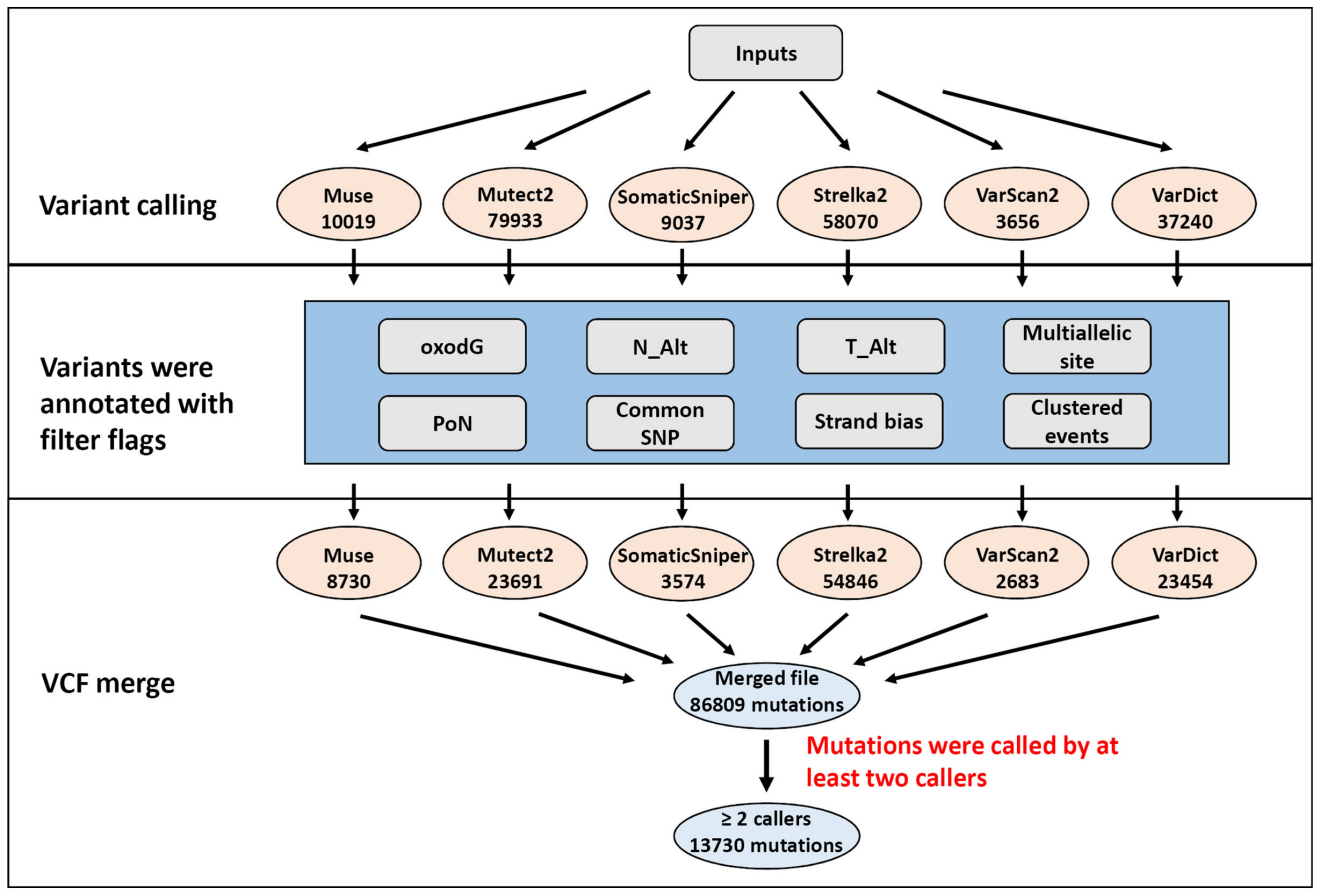
Flowchart of the filtering strategy. Mutations were identified by multiple variant callers parametrized to identify potentially somatic mutations. The number of mutations for each filtering step is depicted in illustration.

**Figure 2:**
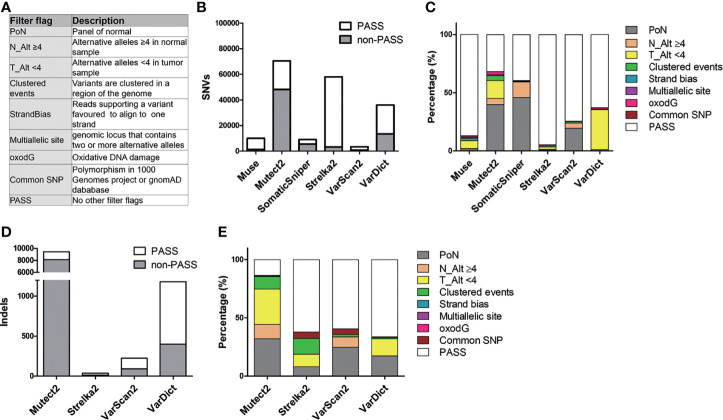
Description of filter flag distribution in six callers. **(A)** Purpose of the filter flags. **(B)** The count of PASS and non-PASS SNVs for each caller. **(C)** The stacked bar chart illustrates the proportion of different filter flags in SNVs for each caller. **(D)** Representation of the amount of PASS and non-PASS indels. **(E)** Histogram of the proportion of different filter flags in indels.

Six different variant calling tools were used to identify somatic mutations in the 50 paired tumor/normal samples: Muse, Mutect2, SomaticSniper, Sterlka2, VarScan2, and VarDict (**Figure 1**). All six callers were used to identify somatic SNVs. Moreover, somatic indels were identified using Mutect2, Strelka2, VarScan2, and VarDict. A total of 163,069 somatic mutations were found by the six callers using the default parameters in the target region (coding and splicing region). Muse, Mutect2, SomaticSniper, Sterlka2, VarScan2, and VarDict detected 10,019, 79,933, 9037, 58,070, 3656, and 37,240 somatic mutations, respectively (**Figure 1**). To reduce false-positive mutations from variant calling, filter flags were used to annotate and assess somatic mutations (**Figure 2A** and **Table S4**). Somatic mutations marked with filter flags, including “common SNP”, “oxodG” oxidative damage, “StrandBias”, “Multiallelic site”, “Clustered events”, and “PoN” variants were then filtered out. Furthermore, somatic mutations that were lacking sufficient evidence to be called a somatic mutation, such as those with low Alt alleles in the tumor sample (T_Alt < 4 alleles) and high Alt alleles in normal samples (N_Alt ≥4 alleles), were filtered out. As result, the filtered (PASS only) data contained 8,730, 23,691, 3,574, 54,846, 2,683, and 23,545 somatic mutations detected by Muse, Mutect2, SomaticSniper, Sterlka2, VarScan2, and VarDict, respectively (**Figure 1** and **Table S4**). As depicted in **Figure S2A**, somatic mutations marked “PASS” filter flag were successfully verified by IGV and Sanger sequencing. However, the somatic mutations which have been labeled “N_Alt ≥4,” “T_Alt < 4,” “Multiallelic site,” and “Clustered event” were not confirmed by Sanger sequencing (**Figures S2B–E**). Furthermore, our results show that all variant callers except Mutect2 and VarDict mis-detected dinucleotide mutations as SNVs. In total, 53 dinucleotide mutations were identified in our study (**Table S5**), all of which were confirmed by Sanger sequencing (**Figure S3**). Finally, the VCF files from each caller were merged (**Figure 1**). Somatic mutations that were only identified by a single caller were excluded. Overall, the ≥2 caller agreeing data set only recovered 8% of the variants in the unfiltered data set. Our study identified 13,730 somatic mutations in 7,729 unique genes, including 3,057 synonymous, 8,541 missense, 17 start loss, 854 stop gain, 9 stop loss, 3 stop retain, 969 splicing site, 53 dinucleotide, 183 frameshift, and 44 in frame mutations (**Figure 1**).

### Effect of Filtering Criteria in Different Callers

**Figure 2** and **Table S6** summarize the process of filtering mutations in the six callers. These variant callers detected a different amount of SNVs and indels (**Figures 2B, D**). For SNVs, Mutect2, Strelka2, and VarDict produced the largest number of unfiltered SNVs (70,532, 58,033, and 36,060, respectively), while Muse, SomaticSniper, and VarScan2 called the smallest number of SNVs (10,019, 9,037, and 3,430, respectively) (**Figure 2B** and **Table S6**). Moreover, Mutect2 and SomaticSniper had high rates of SNVs that did not satisfy the filtering criteria (non-PASS SNVs) (68.2% and 60.5%, respectively), whereas Muse, Strelka2, VarScan2, and VarDict had low rates of non-PASS SNVs (12.9%, 5.5%, 25.7%, and 37.1%, respectively). These non-PASS SNVs mainly consisted of “PoN” and “T_Alt < 4” in Mutect2 (39.9% and 15.1%, respectively) and “PoN” and “N_Alt >4” in SomaticSniper (46.0% and 13.2%, respectively) (**Figure 2C**). For indels, Mutect2 found more unfiltered indels compared to Strelka2, VarScan2, and VarDict (9,401, 37, 226, and 1,180, respectively) (**Figure 2D** and **Table S6**). After filtering, high rates of non-PASS indels were found in Mutect2, whereas Strelka2, VarScan2, and VarDict had low rates of non-PASS indels (86.6%, 37.8%, 40.7% and 33.9%, respectively). These non-PASS indels mainly consisted of “PoN” and “T_Alt < 4” in Mutect2 (32.6% and 30.4%, respectively), “Clustered events” and “T_Alt < 4” in Strelka2 (13.5% and 10.8%, respectively), “PoN” in VarScan2 (24.8%), and “PoN” and “T_Alt < 4” in VarDict (17.4% and 14.7%, respectively) (**Figure 2E**). As such, approximately 23% (37,287/163,069) of unfiltered mutations consisted of PoN variants. Removing mutations marked with the “PoN” filter flag was a crucial step in reducing false-positive rates during WES analysis.

Muse and VarScan2 removed the lowest number of SNVs after filtering with filter flags and removing variants found by a single caller only (**Table S6**). Strelka2 removed the lowest number of indels after variant filtering. Mutect2 removed the largest number of both SNVs and indels after filtering with filter flags and removing variants called by a single caller only. Although SomaticSniper removed a large number of the SNVs selection after filtering with filter flags, it removed a smaller number of SNVs after removing variants called by a single caller. Strelka2 recovered 94.5% of the SNVs after filtering with filter flags but only recovered 19.5% of the same after removing variants called by a single caller. Moreover, Strelka2 found a very small number of indels only.

### Evaluation of Mutation Calling Performance

In order to evaluate the performance of variant calling, IGV was used to visualize and examine somatic mutation in the six callers. The 20 most frequently mutated genes [mutated in at least 7/50 (14%) patients] were utilized for this analysis. **Table S7** shows that the most frequent non-PASS genes during IGV examination were *MUC16*, *MUC19*, *KMT2D*, and *TTN*. To assess the reliability of the IGV examination, a number of mutations for each top 4 IGV non-PASS genes was selected for confirmation by Sanger sequencing. **Figure S4** shows that both IGV-passed and -non-passed mutations could be confirmed by Sanger sequencing. The results of the IGV evaluation were also consistent with those of Sanger sequencing (**Table S8**). In **Table S7**, *MUC16* and *MUC19* genes had a large number of false-positive mutations [2100/2121 (99.0%) and 1900/1906 (99.7%), respectively] that did not satisfy the IGV filtering criteria. The IGV screenshot (**Figure S5**) demonstrates that recurrent false-positive variants were observed at the *MUC16* and *MUC19* loci in our WES data. Therefore, *MUC16* and *MUC19* mutations were removed to reduce false-positive calls. A total of 2276 mutations were retained after removing mutations in *MUC16* and *MUC19*, which then were used to evaluate the validation statistics of the mutation callers.

**Table S9** shows that filtered mutations had significantly higher rates of IGV-PASS compared to unfiltered mutations (*P* < 0.001). The filtered mutations were also associated with a significantly higher rate of IGV-PASS in Mutect2 and SomaticSniper (both *P* < 0.001 and *P* < 0.001). An increased number of callers agreeing on the mutations was associated with an increase in IGV-PASS rates. However, in ≥2, ≥3, ≥4, ≥5 and 6 caller agreeing groups, filtered mutations significantly increased IGV-PASS rates compared to unfiltered mutations. Both filtering with filter flags and selection with mutation calling times were important procedures to reduce false-positive calls.

### The Effect of Read-Depth and Mutation Frequency on Variant Calling

Furthermore, this study examined whether DP and MAF were associated with false-positive variant calls. **Figure S6A** shows the MAF and DP for unfiltered mutations in different mutation calling times. Accordingly, our results show that mutations with high MAF (MAF ≥ 0.8) and high DP (DP ≥ 1000) were mainly distributed in the one caller agreeing group. After filtering with filter flags, the high MAF and high DP mutations were significantly reduced in the one caller group (**Figure S6B**). IGV examination data shows that mutations with high DP (DP ≥ 1000) were mainly distributed in the IGV non-PASS group (**Figure S6C**). The aforementioned data suggest that read depths of 1000 and a MAF of 0.8 can be selected to eliminate false-positives mutations.

Receiver operating characteristic (ROC) curve was used to evaluate the optimal cut-off value of DP and MAF in distinguishing IGV-PASS mutations (**Figures S6E, G**). The AUCs for DP and MAF were 0.478 (95% CI: 0.459–0.496) and 0.607 (95% CI: 0.585–0.628), respectively. At a threshold of 175 reads for DP, the false positive rate and false negative rate were 51% and 45.1% in separating IGV-PASS mutations patients from normal IGV non-PASS mutations. At a threshold of 0.182 for MAF, the false positive rate and false negative rate were 38.5 and 86.3%, respectively. The **Figures S6E, G** showed the relationship between false positive rate and false negative rate with DP or MAF, respectively.

### Estimation of Tumor Mutational Burden in OSCC

**Figure 3:**
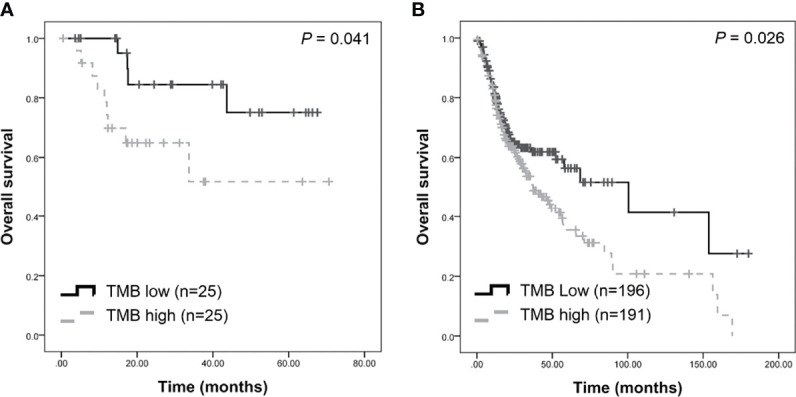
Survival analysis of tumor burden (TMB) in patients with OSCC. Higher TMB were associated with poorer survival in our dataset **(A)** and TCGA-OSCC dataset **(B)**.

TMB was calculated as the number of somatic mutations in the coding region per Mb. In the TCGA-OSCC dataset, 71,890 non-synonymous mutations were identified in 387 patients (185.76 mutations per patient). In our unfiltered data, a total of 163,069 mutations were identified in 50 patients (3261.38 mutations per patient). Our findings show that the mean TMB was 35.81 mutations/Mb per patient. After filtering procedures, 13,730 mutations were retained (274.6 mutations per patient), with the TMB decreasing to 3.01 mutations/Mb per patient in filtered mutations. As depicted in **Table 1**, high TMB was significantly associated with old age (*P* = 0.034) and advanced clinical stage (*P* = 0.021). The median TMB (0.96 mutations/Mb per patient) was considered the cut-off point for assessing outcomes in OSCC. Patients with higher TMB exhibited a poorer outcome compared to those with lower TMB (*P* = 0.041, **Figure 3A**). In TCGA-OSCC dataset, patients with higher TMB also had a poorer outcome compared to those with lower TMB (*P* = 0.026, **Figure 3B**). 

### Genomic Profiling in OSCC

**Figure 4:**
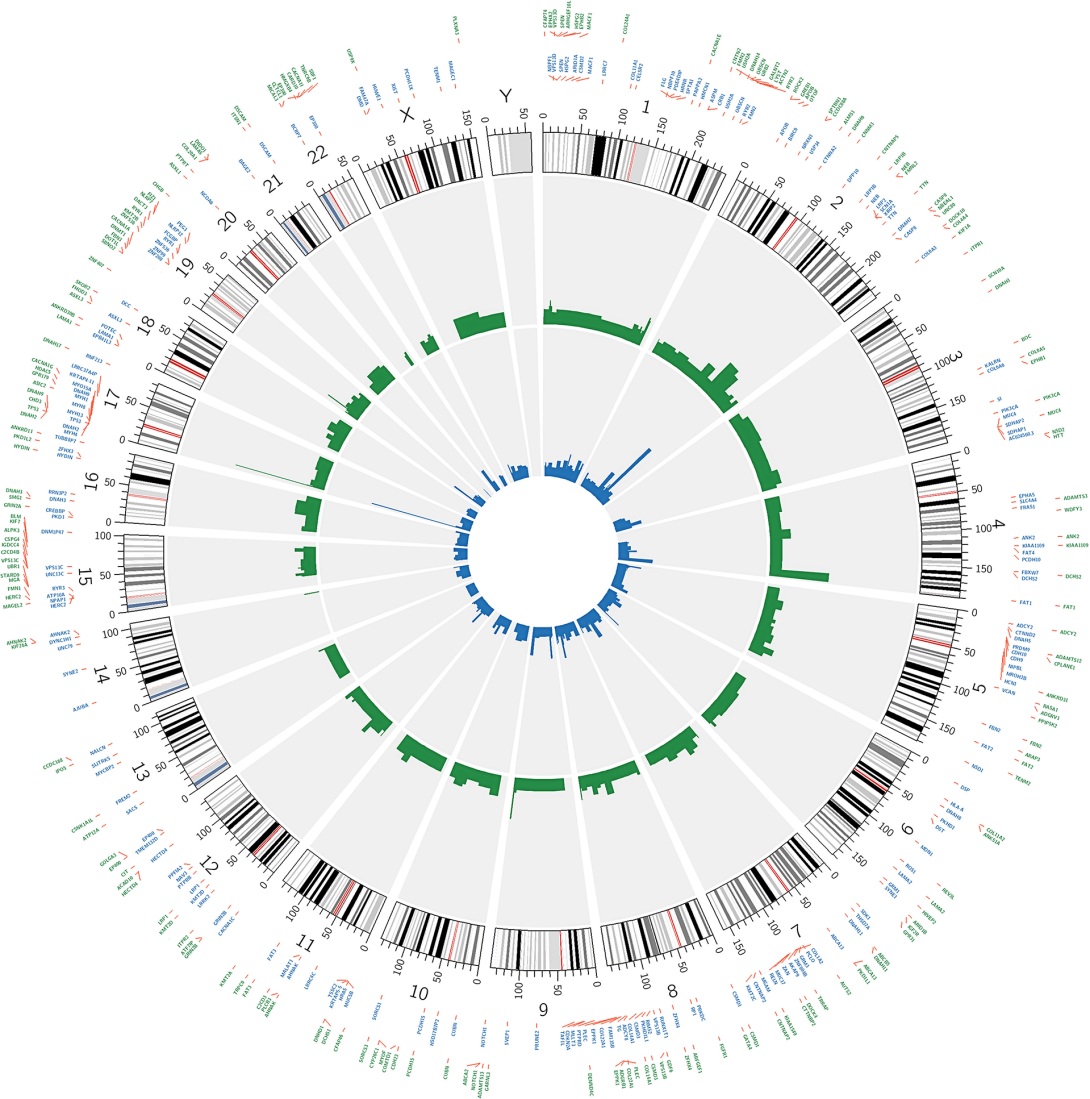
Circos plot showing the 200 most frequently mutated genes in OSCC patients. The inner circle visualizes the mutation frequency of genes in the TCGA OSCC dataset. The outer cycle illustrates the genomic profile which was observed in our study.

CIRCOS plots were used to illustrate the mutational landscape in our study and in the TCGA-OSCC dataset (**Figure 4**). The outer cycle illustrates the genomic profile from our WES data, with the most frequently mutated genes being *TP53* (62%), *FAT1* (40%), *NOTCH1* (28%), and *TTN* (26%) (**Figure 4**). The inner circle illustrates the mutation frequency of the genes in the TCGA-OSCC dataset, with the most mutations observed in *TP53* (68%), *TTN* (42%) *FAT1* (26%), and *CDKN2A* (22%). The mutational landscape in our study appears to be similar to that in the TCGA-OSCC dataset.

We found five novel mutated genes in at least six different patients (≥10%) included herein, which were not detected in the TCGA-OSCC dataset (**Table S10**). The mutation frequencies of *SKOR2*, *CPLANE1*, *CCDC168*, *STARD9*, and *NSD2* were 14%, 12%, 12%, 12%, and 10%, respectively. The InToGene platform and Bailey et al. data sets were used to predict potential mutational driver genes in OSCC. A total of 53 recognized mutational driver genes were found therein, among whom *TP53* and *FAT1* had high mutation rates (**Table S11**). **Table 2** shows the differences in the mutation frequency distribution between our most frequent genes (MAF ≥ 10%) and those in the TCGA-OSCC dataset. Accordingly, among our most frequent genes, 73 genes were identified to have high MAF, whereas only 1 had low MAF. Several of these genes we found were tumor suppressor genes, including *FAT1*, *EPHA2*, *ASXL1*, *PTPRT*, *USP9X*, *IGF2R*, *SPTBN1*, and *PLCB3* (39–43, 45, 47, 48), whereas *ARHGEF10L* and *NSD2* function as oncogenes (44, 55). *TRRAP* has been found to be involved in the regulation of stemness in ovarian cancer stem cells (35), while *DNMT1* and *EPHB1* can regulate tumor progression (38, 46). Other genes have been reported to been involved in the control of proliferation, invasion, and apoptosis in cancer cells (36, 37, 49–54, 56–59).

**Table 2:** List of most frequently mutated genes in our study showed a statistically significant difference in mutation rates compared to TCGA-OSCC dataset.

Genes	Our	TCGA	Driver gene	Involved in cancer^ref^		Genes	Our	TCGA	Driver gene	Involved in cancer^ref^
	MAF	MAF					MAF	MAF		
Our MAF > TCGA MAF						Our MAF > TCGA MAF				
*FAT1*	0.4	0.26	Y	Y (32)		*SBNO2*	0.10	0.02	N	
*RYR2*	0.2	0.09	N			*FBN3*	0.10	0.03	N	
*FMN2*	0.18	0.06	N	Y (35)		*TNRC6B*	0.10	0.02	N	Y (36)
*ABCA13*	0.16	0.07	N			*CFAP74*	0.10	0.01	N	
*EPHA2*	0.16	0.06	Y	Y (33)		*MGA*	0.10	0.03	N	
*HYDIN*	0.16	0.04	N			*CFAP46*	0.10	0.02	N	
*ALPK3*	0.14	0.01	N			*MAGEL2*	0.10	0.02	N	
*ASXL1*	0.14	0.03	Y	Y (34)		*ABCB5*	0.10	0.03	N	Y (37)
*EPPK1*	0.14	0.06	N	Y (38)		*COL24A1*	0.10	0.03	N	
*PKD1L1*	0.14	0.03	N			*SPTBN1*	0.10	0.03	N	Y (39)
*PTPRT*	0.14	0.04	Y	Y (40)		*CLTCL1*	0.10	0.02	N	
*SKOR2*	0.14	#N/A	N			*NSD2*	0.10	#N/A	N	Y (41)
*SORCS3*	0.14	0.05	N			*PLCB3*	0.10	0.02	N	Y (42)
*USP9X*	0.14	0.04	Y	Y (43)		*DNHD1*	0.10	0.02	N	
*WDFY3*	0.14	0.04	N			*KIF26A*	0.10	0.01	N	
*DOCK10*	0.12	0.02	N			*DNMT1*	0.10	0.02	N	Y (44)
*CPLANE1*	0.12	#N/A	N			*COL6A5*	0.10	0.01	N	
*CCDC168*	0.12	#N/A	N			*DCHS1*	0.10	0.03	N	
*ITPR2*	0.12	0.04	N			*C2CD3*	0.10	0.02	N	
*TRRAP*	0.12	0.05	Y	Y (45)		*GOLGA3*	0.10	0.02	N	
*LAMA5*	0.12	0.04	N	Y (46)		*COL20A1*	0.10	0.01	N	
*FBN2*	0.12	0.04	Y			*MICAL3*	0.10	0.03	N	
*STARD9*	0.12	#N/A	N			*BOC*	0.10	0.03	N	
*DNAH1*	0.12	0.04	N			*ADAMTS13*	0.10	0.01	N	
*CACNA1A*	0.12	0.03	N			*CARD10*	0.10	0.02	N	Y (37)
*IGF2R*	0.12	0.03	N	Y (47)		*NBEAL1*	0.10	0.03	N	
*ARHGEF10L*	0.12	0.01	N	Y (48)		*UBR1*	0.10	0.02	N	
*KIF1A*	0.12	0.02	N	Y (49)		*DNAH6*	0.10	0.01	N	
*SCN10A*	0.12	0.03	N			*ZNF407*	0.10	0.02	N	Y (50)
*TRPC6*	0.12	0.02	N	Y (51)		*URB2*	0.10	0.01	N	
*EP400*	0.12	0.04	N			*LYST*	0.10	0.03	N	
*MYOF*	0.12	0.02	N	Y (52)		*ROCK2*	0.10	0.02	N	Y (53)
*SBF1*	0.10	0.02	N	Y (54)		*OTOF*	0.10	0.03	N	
*PPIP5K2*	0.10	0.02	N			*EPHB1*	0.10	0.03	N	Y (55)
*UNC80*	0.10	0.01	N			*AUTS2*	0.10	0.02	N	
*OTOF*	0.10	0.03	N			*DNAH14*	0.10	0.00	N	
Our MAF < TCGA MAF										
*TTN*	0.26	0.47	N					

Fisher’s exact test and chi-square were used as a test for statistical significance.

MAF, minor allele frequency.

Ref, references were cited using superscript numerals.

**Figure 5:**
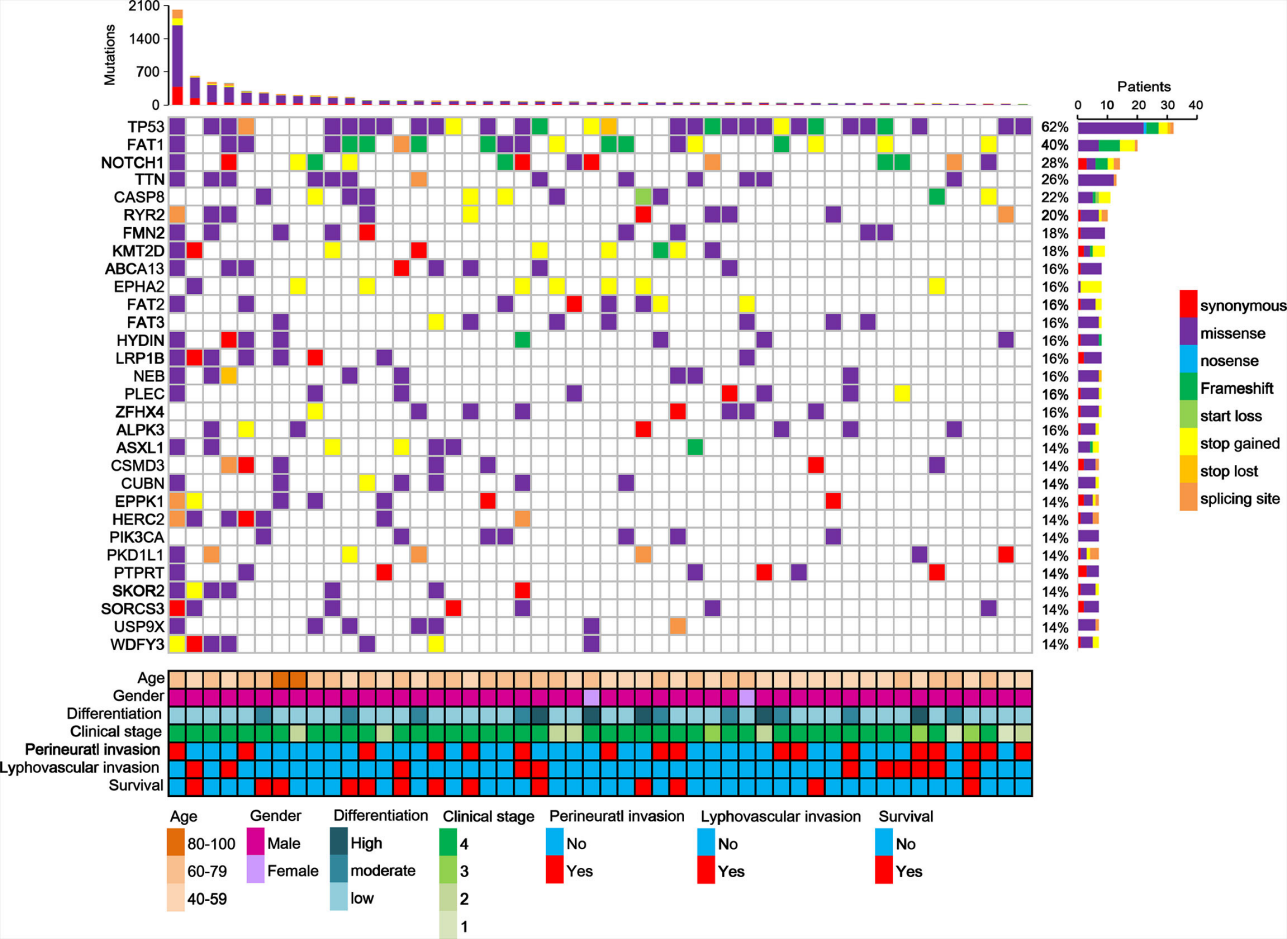
Distribution of the 20 most frequently mutated genes in the OSCC patients. Each column represents an individual OSCC patient, and each row denotes a gene and clinical features. Clinical features and mutation types are color coded as indicated.

**Figure 6:**
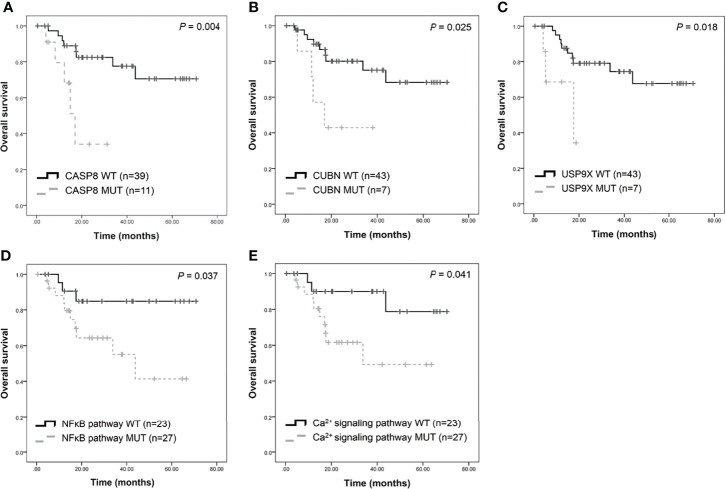
Survival analysis of candidate genes in patients with OSCC. Kaplan-Meier plots of overall survival based on *CASP8* mutation **(A)**, *CUBN* mutation **(B)**, *USP9X* mutation **(C)**, NFκB **(D)**, and calcium signaling pathway **(E)** status.

Furthermore, this study examined the relationship between non-synonymous mutation status and clinical parameters in the 20 most frequently mutated genes (**Figure 5** and **Table S12**). Accordingly, somatic mutation in *ASXL1* was significantly associated with older age (*P* = 0.010) (**Table S12**). In addition, *FAT1* mutations were significantly associated with advanced clinical stage (*P* = 0.033) and marginally significantly associated with T stage (*P* = 0.050). Histological grade was significantly associated with *PKD1L1* mutations (*P* = 0.044) and was marginally significantly associated with *FAT3* mutations (*P* = 0.087). The presence of *TTN* mutations was significantly associated with perineural invasion (*P* = 0.038). Mutation of *FMN2* was marginally significantly associated with T stage (*P* = 0.093) and histological grade mutations (*P* = 0.087). Survival analysis revealed that patients with *CASP8* (log-rank *P* = 0.004), *CUBN* (log-rank *P* = 0.025), and *USP9X* mutations (log-rank *P* = 0.018) had significantly reduced OS rates (**Figures 6A–C**). **Figure S7** showed that distribution of the 20 most frequently mutated genes and clinical features in the TCGA-OSCC patients. In the TCGA-HNSCC dataset, *USP9X* mutation was found to be associated with poor OS (log-rank *P* = 0.010, **Figure S8C**). *CASP8* mutation was marginal significantly associated with OS (log-rank *P* = 0.070, **Figure S8C**). However, there were no significant differences in OS between *CUBN* mutation group and non-*CUBN* mutation group (log-rank *P* =0.885, **Figure S8B**). 

### Molecular Pathway Analysis

**Figure 7:**
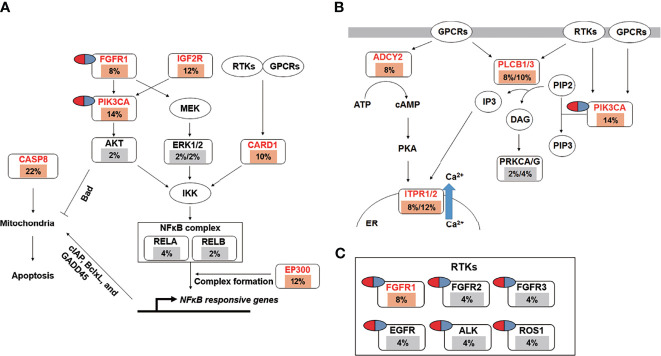
The landscape of major altered genes and significantly altered pathways in OSCC. The NFκB signaling **(A)** and calcium signaling pathways **(B)** were identified by IPA canonical pathway analysis. Pill illustrations show FDA approved drugs which are targeting receptor tyrosine kinase families and *PIK3CA*. Genes were assigned to each pathway and represented with their mutation frequencies (orange font: top 200 frequently mutated genes [≥8%] which were imported into the IPA analysis; gray font: genes with <8% mutation frequencies which were not included in IPA analysis).

Pathway enrichment analysis of the 200 most frequently mutated genes was performed using the IPA. Accordingly, upstream regulator analysis in OSCC (**Figure S9**) showed that *AKT*, *TP53*, and *ERK* were the most predicted upstream regulators controlling different gene clusters in OSCC. **Table 3** shows that four canonical pathways had a *P* value < 0.05 using IPA, including the clathrin-mediated endocytosis signaling, NFκB signaling, PEDF signaling, and the calcium signaling pathways. Furthermore, we evaluated the association between OS and mutations of genes in canonical pathways. Among the patients included herein, 27 (54%) had a mutation in the NFκB signaling-related gene set, including *CARD10*, *CASP8*, *EP300*, *FGFR1*, *IGF2R*, and *PIK3CA* (**Figure 7A**). Mutations in *CARD10*, *CASP8*, *EP300*, *FGFR1*, *IGF2R*, and *PIK3CA* were observed in 5 (10%), 11 (22%), 6 (12%), 4 (8%), 6(12%), and 7 (14%) patients, respectively. Patients with mutations in the NFκB signaling-related gene set had poorer prognosis compared to those without such mutations (**Figure 6D**). However, no significant correlation between mutation status in NFκB signaling-related gene set and OS was found in TCGA-OSCC dataset (**Figure S8D**).

**Table 3:** The canonical pathways of the top 200 frequently mutated genes in HNSCC.

Ingenuity Canonical Pathways	-log (P-value)	Molecules
Clathrin-mediated Endocytosis Signaling	0.856	*APOB*, *CLTCL1*, *EPHB2*, *PIK3CA*, *USP9X*
NFκB Signaling	1.39	*CARD10*, *CASP8*, *EP300*, *FGFR1*, *IGF2R*, *PIK3CA*
PEDF Signaling	1.53	*CASP8*, *PIK3CA*, *ROCK2*, *TP53*
Calcium signaling	1.8	*ADCY2*, *CASP8*, *ITPR1*, *ITPR2*, *PIK3CA*, *PLCB1*, *PLCB3*

PEDF, Pigment epithelium-derived factor.

Mutations in the calcium signaling-related gene set were observed in 27 (54%) patients (**Figure 7B**). Mutations in the calcium signaling-related gene set, including *ADCY2*, *PLCB1*, *PLCB3*, *ITPR1*, *ITPR2*, and *PIK3CA*, were observed in 4 (8%), 4 (8%), 5 (10%), 4 (8%), 6 (12%), and 7 (14%) patients, respectively. Mutations in the calcium signaling-related gene set were associated with poor outcomes in OSCC (**Figure 6E**). In TCGA-OSCC dataset, there was no significant correlation of mutation status in calcium signaling-related gene set with OS (**Figure S8E**).

Thereafter, identified FDA-approved drugs associated with our candidate genes. Accordingly, seven candidate genes were found which may be targeted by FDA-approved drugs. They are involved in the regulation of the NFκB signaling-related and calcium signaling-related pathways (**Figure 7C**). These genes include *PIK3CA* and six receptor tyrosine kinases, namely *FGFR1*, *FGFR2*, *FGFR3*, *EGFR*, *ALK*, and *ROS1* (**Figure 7C**).

## Discussion

False-positive calls are a major problem in the detection of somatic mutations. One of the most effective filters has encoded the expected distribution of alternate allele read counts at every genomic position, based on a large panel of 8000 TCGA normals (PoN) (31). For each genomic position, the “PoN” encodes the distribution of alt read counts across all TCGA normals. This filter tags a somatic variant call if its observed read count is consistent with the “PoN” based on a likelihood test. This allows calls with several supporting reads to be retained when they occur at a site with low allele-fraction (AF) sequencing noise in the “PoN”. To remove high AF artifacts, all somatic calls at a site with recurrently high AF across the “PoN” are removed, whereas those with several supporting reads at the same locus are retained.

Studies have discovered that somatic mutations caused by several carcinogenic and mutagenic chemicals may induce cancer development (60). Somatic, but not germ line, mutations have been found to cause the change from normal cells to cancer cells. and thus being responsible for malignancies (61). Therefore, identifying and removing germ line mutations from the available data is necessary in order to identify true somatic mutations. Aside from comparing paired normal and cancer tissue, another method to differentiate germ line mutations from somatic ones in cancerous tissue is to establish a normal panel. A somatic variant call is tagged by this filter if its observed read count is consistent with the “PoN” based on a likelihood test. Likewise, a common germ line site would have recurring high allelic fractions across the “PoN”. Moreover, a call at that site with a similarly high AF will be flagged.

To remove germ line events or high AF artifacts, approximately 23% of the hits found by mutation calling were initially removed. Different callers had diverse results, with 46.0% and 39.0% of the SNVs not satisfying the filtering criteria in SomaticSniper and Mutect2, respectively. In addition, the Broad Panel of normals flagged almost 30% of the calls in the full set, which were also removed following TCGA data release policies.

There was indeed highly confident evidence for true mutations in our data. Although the ability to call a variant depends on several factors, two key factors include the coverage (or DP) at a site and the frequency of the alternative (i.e. non-reference) allele frequency (18). However, the repeat-rich sequences which are present within centromeric regions and acrocentric short arms, are often fully represented in whole-genome short-read data sets and contribute to inappropriate alignments and high DP signals that localize into a small number of assembled homologous regions (62). Consequently, these regions often provide artifactual peak false-positive calls (62). Efforts to mitigate these mapping errors frequently involve providing an additional ‘decoy’ database or a collection of useful sequences which are missing from the human genome that can help to ensure proper alignment (63). “oxodG” and “Clustered Events” can reduce false-positives (64), with almost 1.9% of the calls in our study having been screened out.

After filtering with “PoN”, “OxodG”, “Clustered Events”, “StrandBias”, “ Multiallelic site”, and “Common SNP”, coverage should no longer be the major factor for false-positive calling. After filtering, IGV non-PASS mutations did not have higher rates of low DP (<100 DP) compared to IGV-PASS mutations (**Figure S6B**). After removing the low AF noise, even an AF of lower than 1% could be identified and validated by the IGV as true mutation.

The possible impact of amplification errors and content bias related to the library method used should nonetheless be considered. Given that potential sources of error may be addressed through assay design, these should be considered early in the design phase of test development (65). Moreover, in cases with rearrangements, isolated neighboring regions may originate from genomic areas which are very distant from the intended or predicted targets. The fragment sizes resulting from shearing and other fragmentation mechanisms will have a considerable influence on the outcome of the analysis. Shorter fragments will be captured with higher specificity than longer fragments, as the former will contain a lower proportion of off-target sequences. On the other hand, longer reads are expected to map to the reference sequence with less accordance than shorter reads (66). As such, several large genes, including *TTN*, *MUC19*, *MUC16*, and *KMT2D*, had more false-positive results that could be screened by our study.

Despite removing the normal panel SNPs and repeat-rich sequences, false-positive mutations still are present (31). Thus, combining the results of several mutation callers is important to reduce false-positives while maintaining sensitivity (18), which is helping with the identification of true somatic mutations. However, this approach is raising some questions, such as how many mutation callers should be utilized. Analyzing the number of true and false detections in any combination of mutation callers across all replicates suggests that the combination of at least of two callers has a significantly better performance compared to individual callers (18, 31). Fortunately, intersecting mutation callers did not diminish the amount of the identified true mutations. Karimnez found only 1 out of 343 gold-standard mutations was missing, when intersectioning five programs (18). Comparing our intersectioning results with those of other WES studies and the TCGA database yielded differences in the main mutated genes and in the mutated allele frequencies (**Table 2**). We conducted IGV and Sanger sequencing to demonstrate that our data was more precise in detecting true mutations (**Table S8**).

TMB is defined as the total number of somatic gene coding errors, base substitutions, insertions, or deletions per megabase of tumor tissue (67). According to the studies conducted previously, the estimated TMB value for each sample is defined as the total mutation frequency divided by the length of the combined human exons (38 Mb) (68–70). We determined the TMB in patients with tumor and blood samples sufficient for WES (71, 72). Different algorithms have been found to produce varying results regarding the tumor burden. Accordingly, in several studies, the TMB of OSCC ranged from 4 to 104 mutations (7, 8, 10, 73, 74), whereas others have found a TMB of 2.079 (68), 2.96 (69), and 4.7 (70), mutations when the mutation count was divided by the length of the complete human exon data (38 Mb). We therefore suggest to use the target region’s true length when counting the mutation burden (i.e., the SureSelect Human All Exon v6 + UTR 91.08 Mb in the current study) to indicate the true burden. Given that the total size of the WES was not uniform across various studies, using 38 MB as the denominator to calculate the TMB from the TCGA database will incur bias distortion. As such, our data evaluates the mutation burden more precisely, which then can be correlated with clinical parameters, including disease stage and OS rate of OSCC. Interestingly, age has been found to be only marginally related to TMB. Nonetheless, more samples may be collected and analyzed for further elucidation.

Several studies have identified landscape mutations in HNSCC (7, 8, 13, 14, 34, 75–80). When somatic variant callers were first compared, a surprisingly large number of unique calls was identified for each method (17). Recently, a few papers have used a combination of Mutect2 and Strelka to screen for mutations (13, 77, 80). Accordingly, Nisa used Mutect and VarScan2 to identify mutation patterns between metastatic and recurrent HNSCC (79). Nonetheless, there are still differences in the callers, parameters, and filters which were used in the different projects (31). Ideally, future variant calling and filtering efforts should use robust benchmarking to evaluate various combinations of callers, filters, and parameters and determine which callers and filters are optimal for OSCC (31). The study presented here used multiple pipelines to call mutations. The combination of all six programs was able to identify 98.6% of the actual mutations but resulted in a 50% loss of point mutations. After filtering and combination, two different programs were found to achieve a specificity of 88.7%.

Using the intersection of multiple mutation calling pipelines helped to identify true novel somatic mutations, including *SKOR2*, *CCDC168*, *STARD9*, and *CFAP74*, with allele frequencies of 0.14, 0.12, 0.12, and 0.1. Still, the verification of this pathway requires further evaluation.

The effectiveness of different therapeutic modalities is largely dependent on the mutational profile of a tumor, given that genetic alterations are likely to confer new oncogenic potential to cancer cells (81). The precise targeting of these alterations, together with a modifications of the treatment regimen, decreases therapeutic resistance, possibly saving countless patients from morbidity and mortality. Modern research has unveiled a new mutational landscape for oral cancer and factors contributing to the resistance and therapeutic efforts (2). The p53 and RB pathways are playing a key role in cell cycle control and have been found to be frequently abrogated in HPV−negative tumors, which may then be followed by the activation of PI3K pathway (2). No mutually exclusive or concordant mutations exist with those in *PIK3CA*. FAT1–NOTCH1–AJUBA pathway alterations that impact β-catenin signaling might form another route through which carcinogenesis is triggered. The role of *EGFR* and other growth factor receptors and their ligands warrants further study with regard to inappropriate models by genome editing, although some data suggest that amplification or mutation of these proteins may serve as an alternative to *CCND1* amplification (2). However, several genes are mutated at only very low frequencies with often unclear functional consequences. Using an intersectional analysis for mutation calling, the mutation pattern is not completely like reported before. *APOB*, *CLTCL1*, *EPHB2*, *PIK3CA*, and *USP9X* are involved with clathrin-mediated endocytosis (CME) signaling. CME is responsible for the uptake of transmembrane receptors and transporters, remodeling the plasma membrane composition in response to environmental changes, and regulating cell surface signaling (40). Studies have found that EGF-dependent cell proliferation is enhanced in CME-defective cells (82). Mutant *USP9X* has been found to be associated with poor prognosis. As well, mutations in *ADCY2*, *CASP8*, *ITPR1*, *ITPR2*, *PIK3CA*, *PLCB1*, and *PLCB3* (which are involved in calcium signaling) are associated with poor prognosis. These genes may be targeted by new compounds to find out if the can improve OSCC prognosis.

A confident caller with a low false-positive profile is better suited for the discovery of driver genes, given that the removal of false-positive noise is helping researchers with the identification of significantly recurring patterns. Once the significant driver genes have been identified, a second pass over the mutation data set can identify calls of lower confidence that could provide additional examples of the gene of interest. Our results show that several genes, including *ASXL*, *FAT1*, *PKD1L1*, *TTN*, *FAT3*, and *FMN2*, are associated with clinicopathological parameters.

Although the MC3 program produced high-quality calls within each tumor-specific analysis group, differences in the callers, parameters, and filters used still were present from one project to another (31). Ellrott et al. used multiple genomic pipelines to identify mutation calling of tumor exomes by building standardized genomic analysis pipelines which can be massively deployed to tens of thousands of samples. However, care should be taken when analyzing a wide variety of cohorts (31). 

## Conclusions

After performing the identification of mutations with an array of six mutation callers adopted by different analysis centers, this study demonstrates that consensus calling outperformed single algorithms both regarding sensitivity and validation status. Finally, the use of consistent methods for calling enhances the utility of this resource in future endeavors to compare the molecular makeup across different studies. The results of this effort provide the integral components which are necessary for future studies in somatic variant calling. 

## Data Availability Statement

The datasets presented in this study can be found in online repositories. The names of the repository/repositories and accession number(s) can be found below: https://www.ncbi.nlm.nih.gov/genbank/, BankIt2445141. 

## Ethics Statement

The studies involving human participants were reviewed and approved by Mackay Memorial Hospital. The patients/participants provided their written informed consent to participate in this study. 

## Author Contributions

Conception: C-JL. Lab work: H-WC and C-HC. Interpretation or analysis of data: L-HL, C-JL, and C-HC. Preparation of the manuscript: L-HL, K-WC, and C-JL. Revision for important intellectual content: C-JL. Supervision: C-JL and K-WC. All authors contributed to the article and approved the submitted version.

## Funding

This study was supported by grants from MacKay Memorial Hospital (MMH-E-105-12 and MMH-E-108-12) and the Ministry of Science and Technology, Taiwan (MOST 108-2314-B-195 -002 -MY2 and MOST 105-2314-B-195-005-MY3). 

## Conflict of Interest

The authors declare that the research was conducted in the absence of any commercial or financial relationships that could be construed as a potential conflict of interest.

## Publisher’s Note

All claims expressed in this article are solely those of the authors and do not necessarily represent those of their affiliated organizations, or those of the publisher, the editors and the reviewers. Any product that may be evaluated in this article, or claim that may be made by its manufacturer, is not guaranteed or endorsed by the publisher.

## Supplementary Material

Supplementary Figure 1 Somatic mutation calling pipelines. Aligned data were analyzed by six differently callers to generate VCF files. VCF files were further filtered out sequence context artefacts and germline variants.

Supplementary Figure 2 Validation of filter flags by Sanger sequencing and IGV. Variants that were marked with (A) “PASS”, (B) “N_Alt”, (C) “T_Alt”, (D) “Multiallelic site”, and (E) “Cluster event” were examined by direct sequencing and IGV (screenshot). Except for PASS variants, variants which had been marked with other filter flags were considered to be false positive variants.

Supplementary Figure 3 Confirmation by Sanger sequencing of dinucleotide mutations called by Mutect2. Sanger sequencing and IGV screenshot of dinucleotide mutations in **(A)** *FAN1* chr15:30925801_GG>TT and **(B)** *RAI1* chr17:17798486_GG>CA.

Supplementary Figure 4 Validation of IGV filtering results by Sanger sequencing. IGV PASS variants were consistent with those of the Sanger sequencing in **(A)** *MUC16*, **(C)** *MUC19*, and **(E)** *KMT2D*. IGV non-PASS variants were not detected by Sanger sequencing in **(B)** *MUC16*, **(D)** *MUC19*, and **(F)** *KMT2D*. Solid arrows indicate the positions of the mutations. Open arrows indicate the positions of polymorphisms.

Supplementary Figure 5 Recurrent false positives in *MUC16* and *MUC19*. In the zoomed-out pane, IGV visualizes that both in normal and tumor samples, a large number of variants were detected in (A) the *MUC16* and (B) the *MUC19* locus. These observations were not only made in in patients 1074 and 700 but also in other patients.

Supplementary Figure 6 Association between mutation frequency and read depth with false positive variant calls. Distribution of mutation frequency and read depth in (A) unfiltered data, (B) filtered data, and (C) IGV examination data. Receiver operating characteristic (ROC) curve was performed to evaluate the threshold value of DP **(D)** and MAF **(F)** in distinguishing IGV-PASS mutations. Distribution of false positive rate (blue) and false negative rate (red) base on DP **(D)** or MAF **(F)** values.

Supplementary Figure 7 Distribution of the 20 most frequently mutated genes in the TCGA-OSCC patients. Each column represents an individual OSCC patient, and each row denotes a gene and clinical features. Clinical features and mutation types are color coded as indicated. Data were extracted from the TCGA database (https://portal.gdc.cancer.gov/).

Supplementary Figure 8 Survival analysis of candidate genes in patients with TCGA-OSCC. Kaplan-Meier plots of overall survival based on *CASP8* mutation **(A)**, *CUBN* mutation **(B)**, *USP9X* mutation **(C)**, NFκB **(D)**, and calcium signaling pathway **(E)** status.

Supplementary Figure 9 Regulator Effect networks identified by IPA in top 200 frequently mutated genes. We imported the 200 most frequently mutated genes into IPA, these genes were mutated in at least 7 patients (highlighted in blue). **(A)** *Akt* **(B)**, *TP53*, and **(C)** *ERK1/2* were located in the core that is the most important factor in our dataset. The red frame indicates that the genes were associated with survival outcomes in the Kaplan-Meier analysis.
