# Supplementary figures and images for: Precise Identification of Recurrent Somatic Mutations in Oral Cancer Through Whole-Exome Sequencing Using Multiple Mutation Calling Pipelines
Source: Front Oncol. 2021 Nov 29;11:741626. doi: 10.3389/fonc.2021.741626 (PMC8666431; doi:10.3389/fonc.2021.741626)

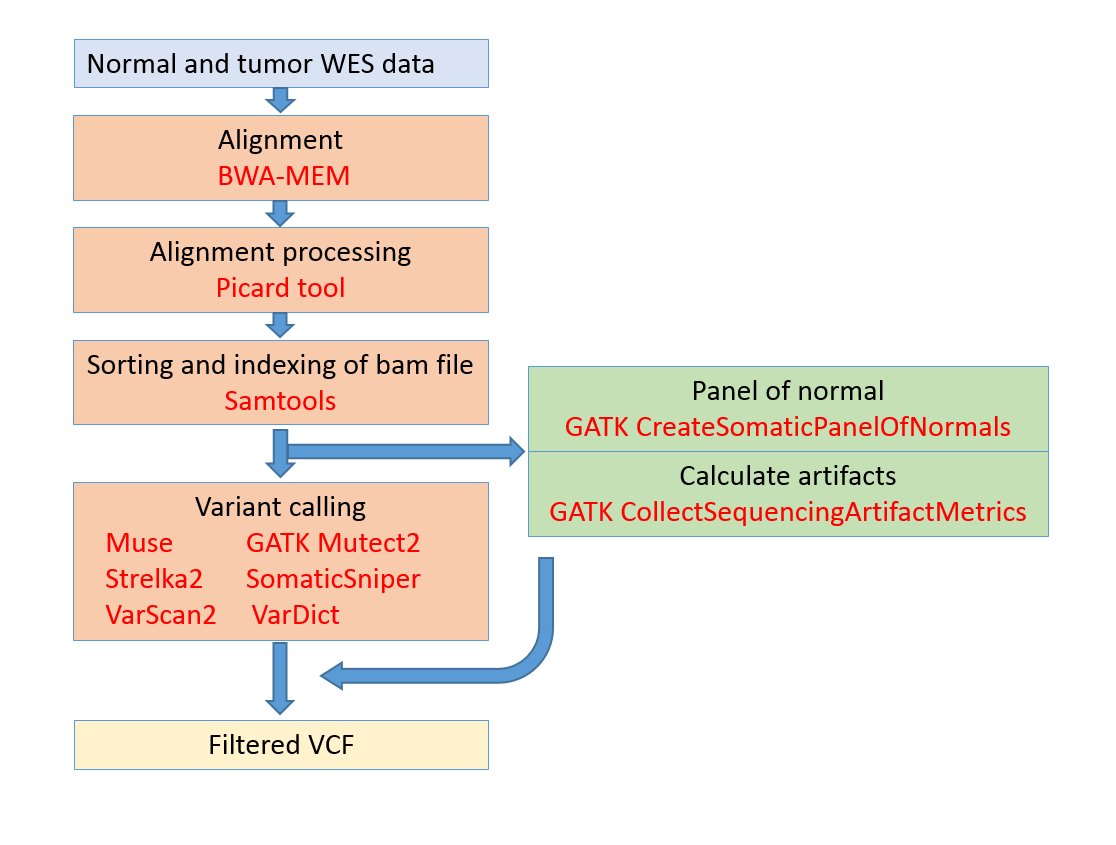

Supplement: Supplementary Figure 1 — Somatic mutation calling pipelines. Aligned data were analyzed by six differently callers to generate VCF files. VCF files were further filtered out sequence context artefacts and germline variants. [file Image_1.tif]

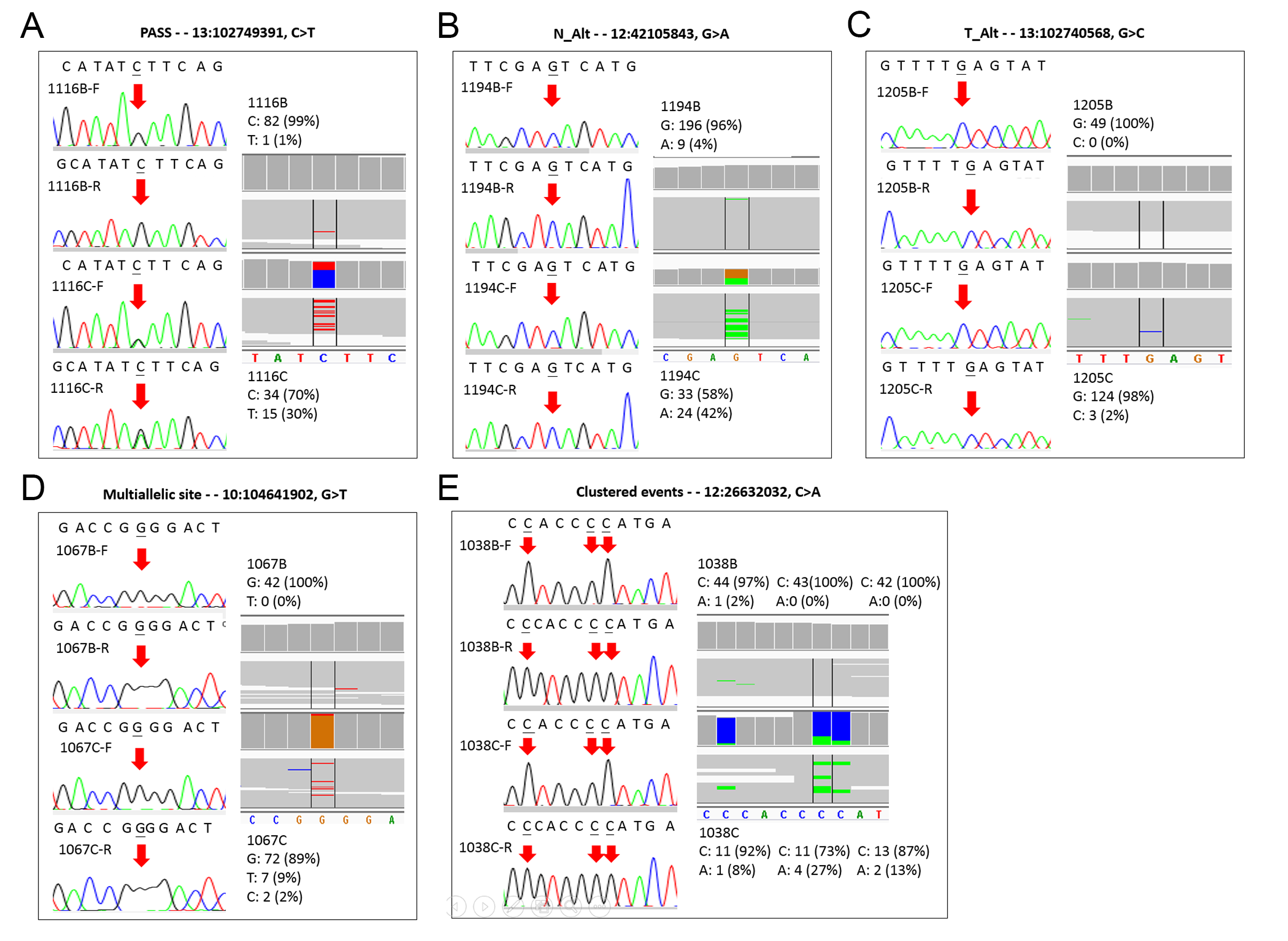

Supplement: Supplementary Figure 2 — Validation of filter flags by Sanger sequencing and IGV. Variants that were marked with (A) “PASS”, (B) “N_Alt”, (C) “T_Alt”, (D) “Multiallelic site”, and (E) “Cluster event” were examined by direct sequencing and IGV (screenshot). Except for PASS variants, variants which had been marked with other filter flags were considered to be false positive variants. [file Image_2.tif]

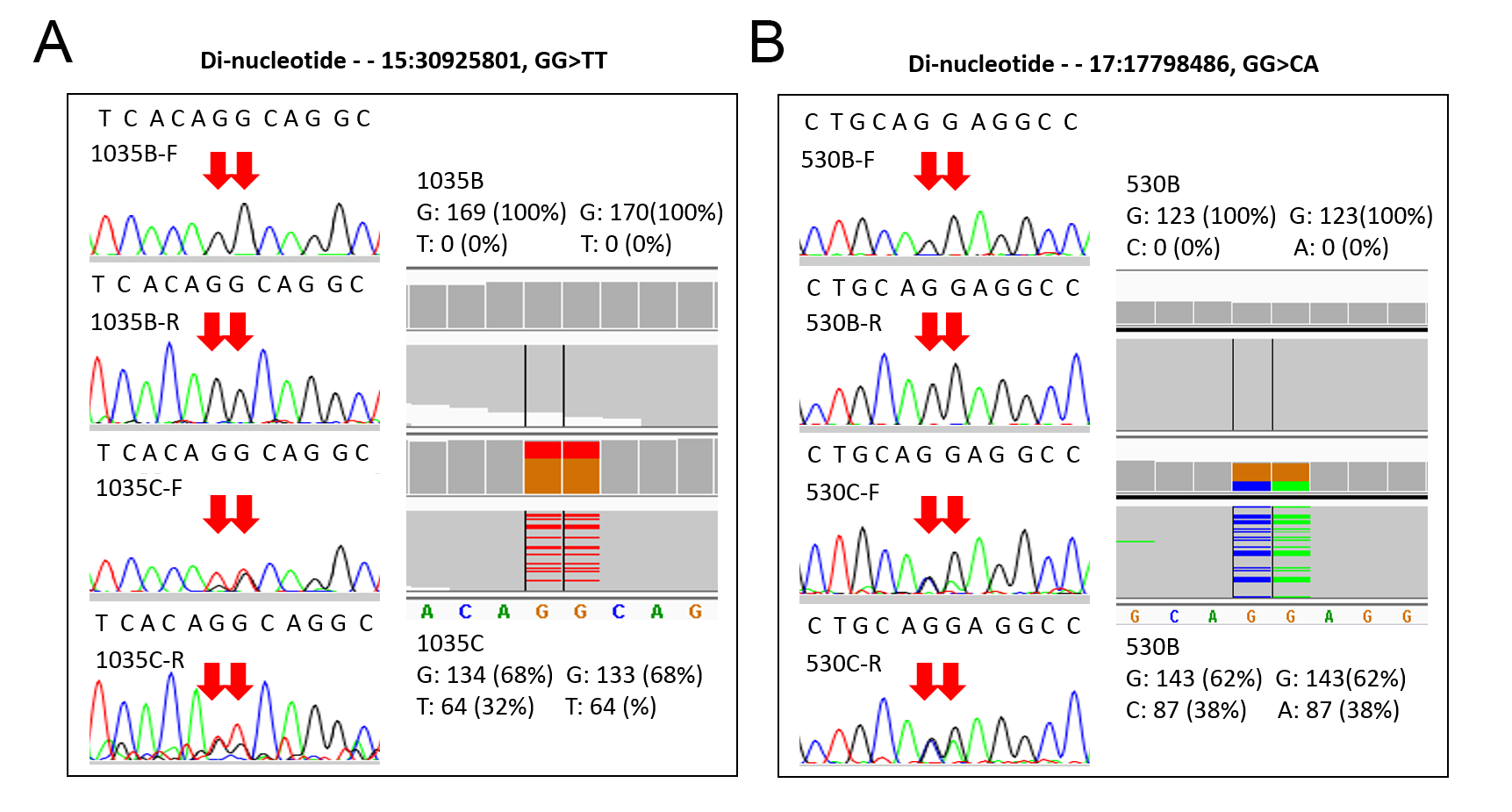

Supplement: Supplementary Figure 3 — Confirmation by Sanger sequencing of dinucleotide mutations called by Mutect2. Sanger sequencing and IGV screenshot of dinucleotide mutations in (A) FAN1 chr15:30925801_GG>TT and (B) RAI1 chr17:17798486_GG>CA. [file Image_3.tif]

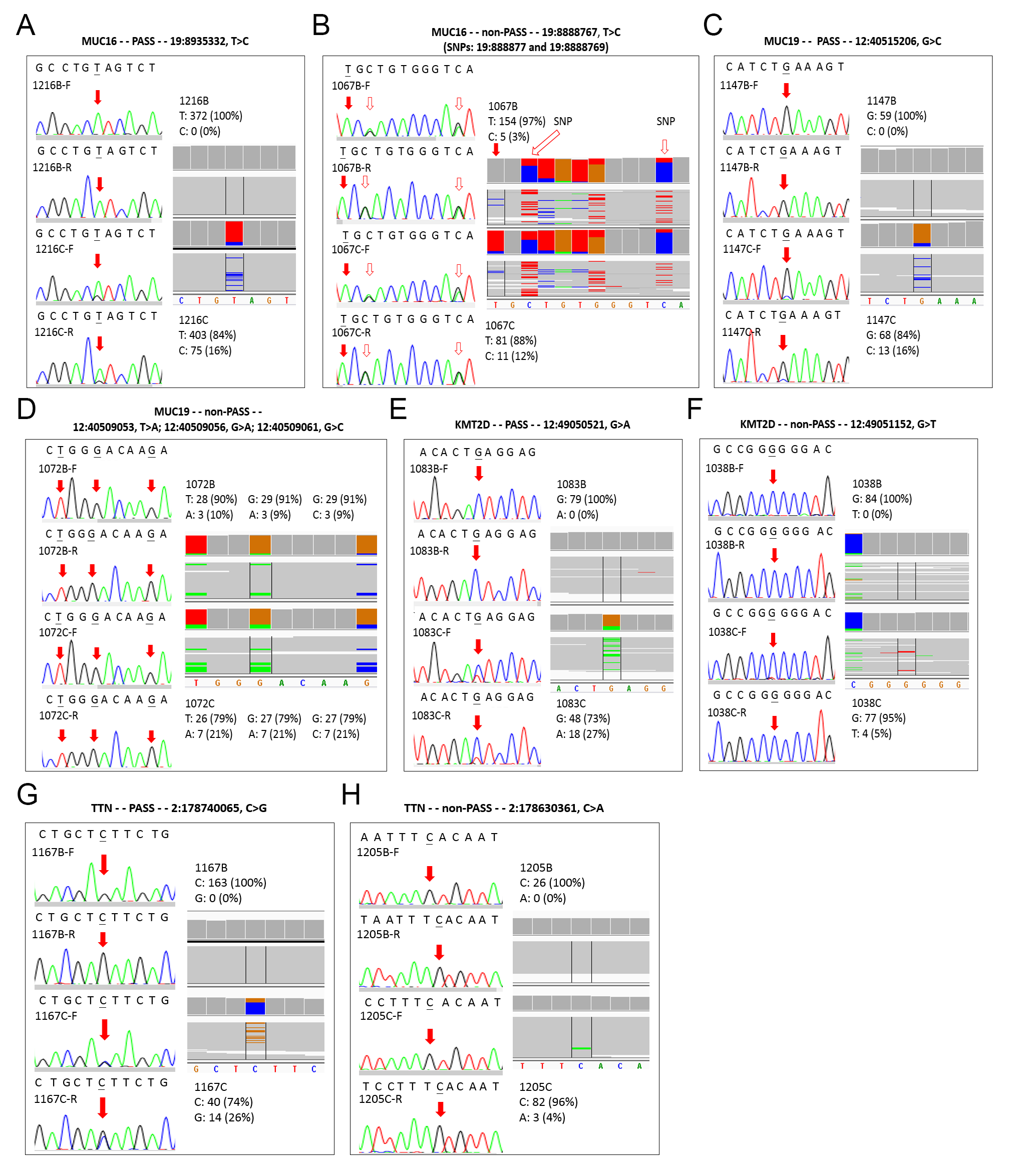

Supplement: Supplementary Figure 4 — Validation of IGV filtering results by Sanger sequencing. IGV PASS variants were consistent with those of the Sanger sequencing in (A) MUC16, (C) MUC19, and (E) KMT2D. IGV non-PASS variants were not detected by Sanger sequencing in (B) MUC16, (D) MUC19, and (F) KMT2D. Solid arrows indicate the positions of the mutations. Open arrows indicate the positions of polymorphisms. [file Image_4.tif]

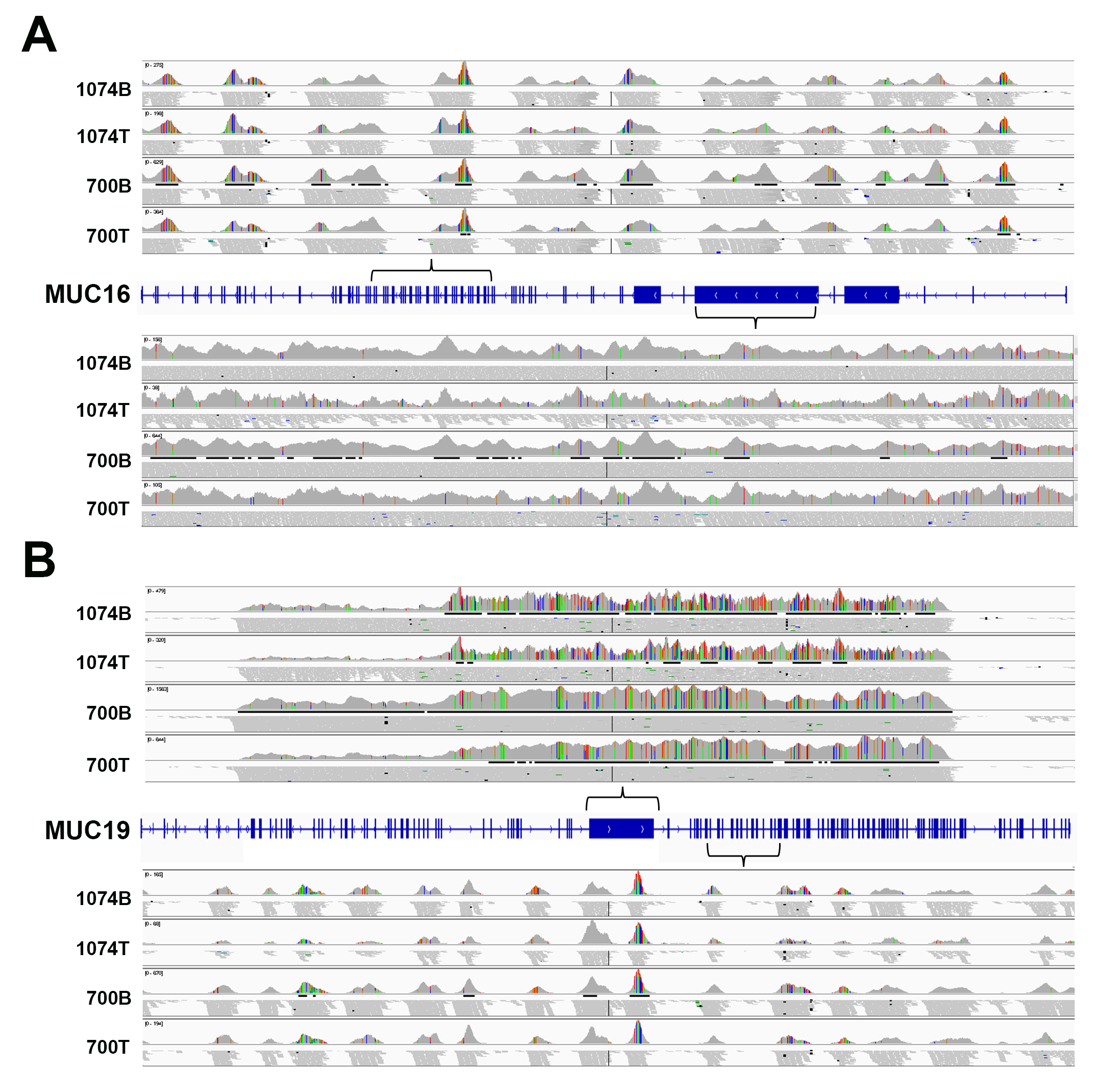

Supplement: Supplementary Figure 5 — Recurrent false positives in MUC16 and MUC19. In the zoomed-out pane, IGV visualizes that both in normal and tumor samples, a large number of variants were detected in (A) the MUC16 and (B) the MUC19 locus. These observations were not only made in in patients 1074 and 700 but also in other patients. [file Image_5.tif]

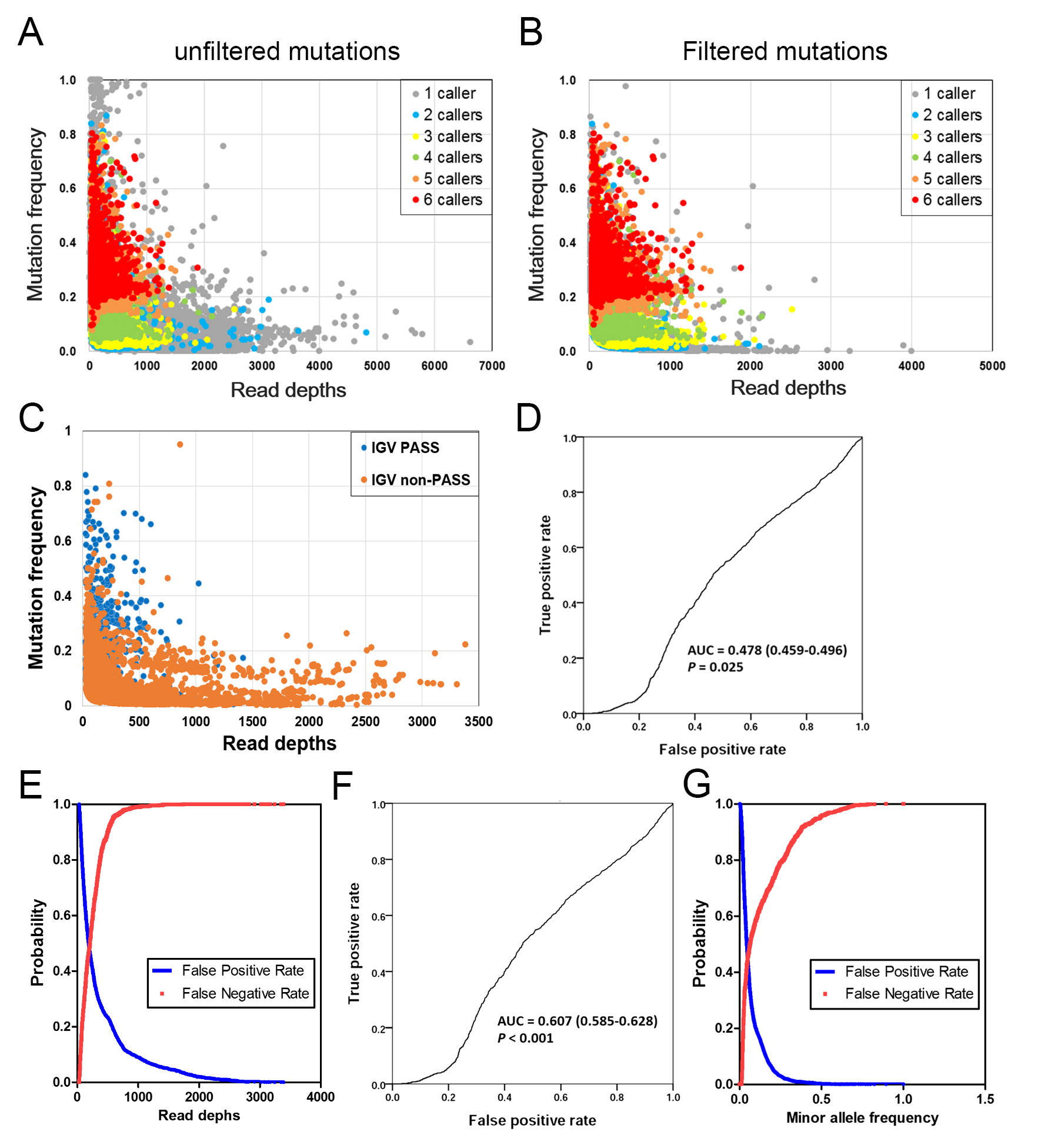

Supplement: Supplementary Figure 6 — Association between mutation frequency and read depth with false positive variant calls. Distribution of mutation frequency and read depth in (A) unfiltered data, (B) filtered data, and (C) IGV examination data. Receiver operating characteristic (ROC) curve was performed to evaluate the threshold value of DP (D) and MAF (F) in distinguishing IGV-PASS mutations. Distribution of false positive rate (blue) and false negative rate (red) base on DP (D) or MAF (F) values. [file Image_6.tif]

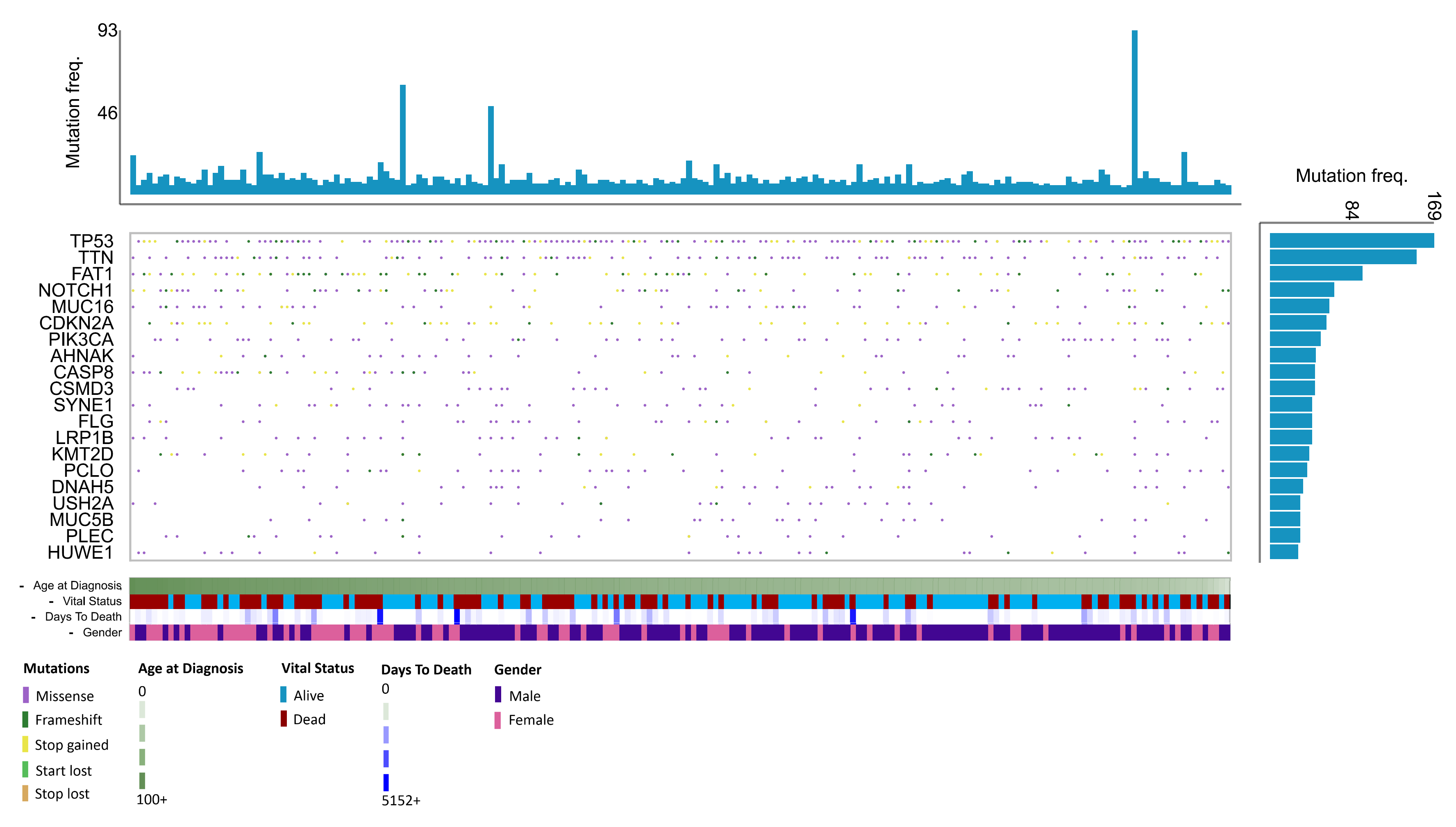

Supplement: Supplementary Figure 7 — Distribution of the 20 most frequently mutated genes in the TCGA-OSCC patients. Each column represents an individual OSCC patient, and each row denotes a gene and clinical features. Clinical features and mutation types are color coded as indicated. Data were extracted from the TCGA database (https://portal.gdc.cancer.gov/). [file Image_7.tif]

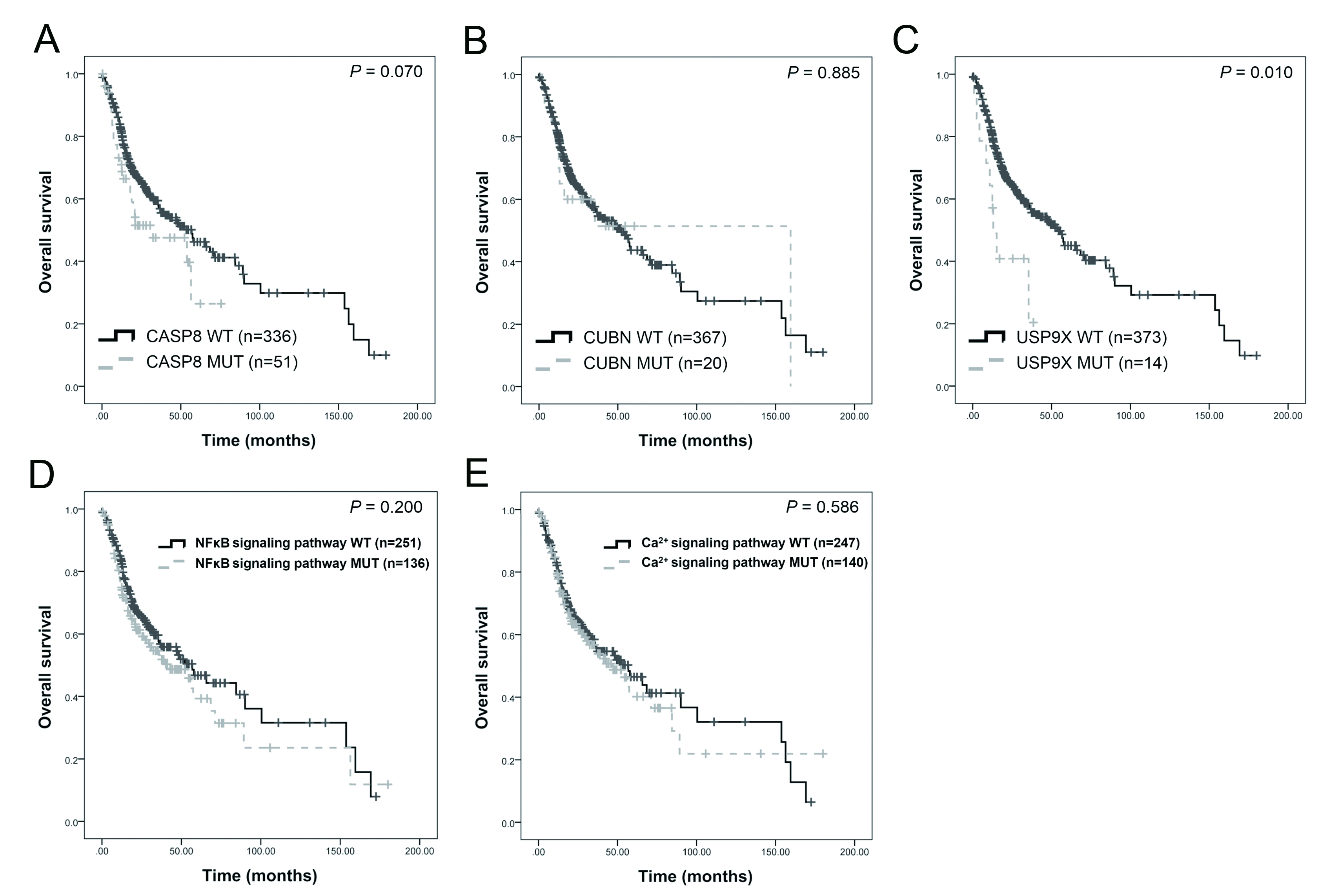

Supplement: Supplementary Figure 8 — Survival analysis of candidate genes in patients with TCGA-OSCC. Kaplan-Meier plots of overall survival based on CASP8 mutation (A), CUBN mutation (B), USP9X mutation (C), NFκB (D), and calcium signaling pathway (E) status. [file Image_8.tif]

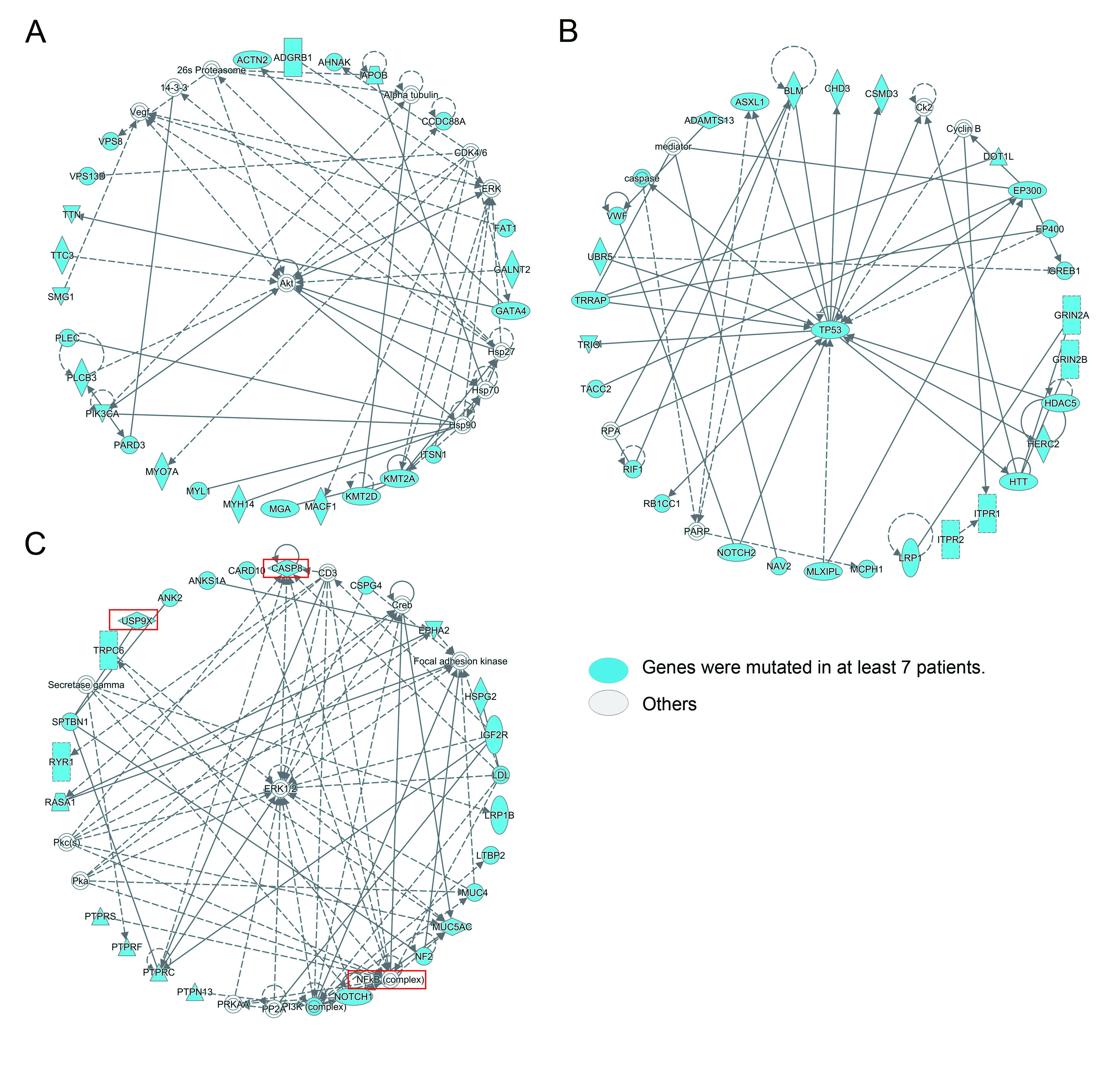

Supplement: Supplementary Figure 9 — Regulator Effect networks identified by IPA in top 200 frequently mutated genes. We imported the 200 most frequently mutated genes into IPA, these genes were mutated in at least 7 patients (highlighted in blue). (A) Akt (B), TP53, and (C) ERK1/2 were located in the core that is the most important factor in our dataset. The red frame indicates that the genes were associated with survival outcomes in the Kaplan-Meier analysis. [file Image_9.jpeg]
