# Supplementary material for: Precise Identification of Recurrent Somatic Mutations in Oral Cancer Through Whole-Exome Sequencing Using Multiple Mutation Calling Pipelines
Source: Front Oncol. 2021 Nov 29;11:741626. doi: 10.3389/fonc.2021.741626 (PMC8666431; doi:10.3389/fonc.2021.741626)
Supplement: Supplementary file 10 [file Table_1.docx]

| Table S1. The software used to analyze whole-exome sequencing data | | |
| --- | --- | --- |
| Software and Algorithms | Version | SOURCE |
| BWA-MEM | 0.7.15 | <http://bio-bwa.sourceforge.net/> |
| SAMtools | 1.3.1 | <http://samtools.sourceforge.net/> |
| Muse | 1.0rc | <https://bioinformatics.mdanderson.org/public-software/muse/> |
| SomaticSniper | 1.0.5.0 | <http://gmt.genome.wustl.edu/packages/somatic-sniper/> |
| Strelka2 | 2.9.10 | <https://github.com/Illumina/strelka/> |
| VarScan2 | 2.4.3 | <http://varscan.sourceforge.net/> |
| VarDict | 1.8.2 | https://github.com/AstraZeneca-NGS/VarDictJava |
| GATK tools: | 4.1.0.0 | <https://gatk.broadinstitute.org/hc/en-us/> |
| Mutect2 |  |  |
| CreateSomaticPanelOfNormals  CollectSequencingArtifactMetrics |  |  |
| FilterByOrientationBias |  |  |
| CalculateContamination |  |  |
| GetPileupSummaries |  |  |
| FilterMutectCalls |  |  |

| Table S2. List the command line of six variant calling tools | |
| --- | --- |
| Algorithms | Command line |
| Muse | ./MuSE call -r region -f hg38.fa Tumor.bam Normal.bam -O Output.Prefix ./MuSE sump -I Output.Prefix.MuSE.txt -E -O Output.vcf |
| Mutect2 | gatk --java-options -Xmx64G Mutect2 -R hg38.fa -I Tumor.bam -I Normal.bam -normal Normal tumor Tumor --germline-resource af-only-gnomad.hg38.vcf --panel-of-normals somatic_1000g_pon.hg38.vcf -O output.vcf --native-pair-hmm-threads 16 --tumor-lod-to-emit 3.0 --independent-mates true |
| SomaticSniper | ./bam-somaticsniper -G -L -F vcf -f hg38.fa Tumor.bam Normal.bam Output.vcf |
| Strelka2 | ./configManta.py --bam=Normal.bam --tumorBam=Tumor.bam --referenceFasta=$BWAIndex --runDir ./SV/ ./SV/runWorkflow.py -m local -j 16  ./configureStrelkaSomaticWorkflow.py --normalBam=Normal.bam --tumorBam=Tumor.bam --indelCandidates=./SV/results/variants/candidateSmallIndels.vcf.gz --referenceFasta=hg38 --runDir=./SNV/ ./SNV/runWorkflow.py -m local -j 16 |
| VarScan2 | samtools mpileup -q 1 -f hg38.fa Tumor.bam > Tumor.mpileup samtools mpileup -q 1 -f hg38.fa Normal.bam > Normal.mpileup java -jar VarScan.v2.4.3.jar somatic Normal.mpileup Tumor.mpileup Tumor --output-vcf Output.vcf |
| VarDict | ./VarDict th 16 -G hg38.fa -f 0.001 -N Tumor -b "/path/Tumor.bam\|paht/Normal.bam" -c 1 -S 2 -E 3 -g 4 nextera_region.bed \| testsomatic.R \| var2vcf_paired.pl -N Tumor\|Normal -f 0.001 > Output.vcf |

| Table S3. Primer list for PCR and Sanger sequence | | | | |
| --- | --- | --- | --- | --- |
| Primer name | Target | Variant types | Tm (℃) | Sequence |
| 13:102749391 | *CCDC168* | SNV | 56 | F:5'-AGGTGAGGACCAACATTGTG-3' |
|  |  |  |  | R:5'-TGCCCTTTAATGGTAGACAAAA-3' |
| 13:102740568 | *CCDC168* | SNV | 56 | F:5'-AAAAATTGAGGATGAAGCATCT-3' |
|  |  |  |  | R:5'-TGCTCCCAAAAGTTCTCATAGG-3' |
| 12:42105843 | *GXYLT1* | SNV | 60 | F:5'-TGGTTTGTTTTCATATCTTCTCTGA-3' |
|  |  |  |  | R:5'-TCCATTGTCATGTCATCTGGTT-3' |
| 12:26632032 | *ITPR2* | SNV | 56 | F:5'-GCCCAAACAATGCTTATGCT-3' |
|  |  |  |  | R:5'-ACAGTCACATCCTCGTGCTC-3' |
| 10:104641902 | *SORCS3* | SNV | 66 | F:5'-GACGGGGCTCCTACTCTTGT-3' |
|  |  |  |  | R:5'-GTGGGTACTCACGCTGCTG-3' |
| 15:30925801 | *FAN1* | Di-nucleotide | 60 | F:5'-AGGGAGGTCAATCCTCACG-3' |
|  |  |  |  | R:5'-AAAACCGCTGCGTCTGTAAT-3' |
| 17:17798486 | *RAI1* | Di-nucleotide | 63 | F:5'-AGCTGCTACTGCTGTGATGG-3' |
|  |  |  |  | R:5'-AGACACATTTGGCCCACACT-3' |
| MUC19-PASS1 | *MUC19* | SNV | 60 | F:5'-TGGAAGGATCAGAGGCAACT-3' |
|  |  |  |  | R:5'-TGTCTCTCCAGCTGATGGTC-3' |
| MUC19-PASS2 | *MUC19* | SNV | 58 | F:5'-CAGGCCAGGTAGGAAGGACT-3' |
|  |  |  |  | R:5'-TGGCATTACCCCCTAAATCA-3' |
| MUC19-nonPASS1 | *MUC19* | SNV | 58 | F:5'-AAATAGCCAGCAGCAAAACAA-3' |
|  |  |  |  | R:5'-GCCGCTGAGAAAAGAAAGAA-3' |
| MUC19-nonPASS2 | *MUC19* | SNV | 58 | F:5'-GCCCAGTTTCCTGAGATATTG-3' |
|  |  |  |  | R:5'-TGGCCTCTGAGAGAAAAGAAA-3' |
| KMT2D-PASS/nonPASS | *KMT2D* | SNV | 60 | F:5'-CTGTCTCCTTTGGGGGAGTT-3' |
|  |  |  |  | R:5'-AAACTGCCAGGGGTCTGTC-3' |
| KMT2D-nonPASS2 | *KMT2D* | SNV | 58 | F:5'-CTATGCGCTGTGCCTGAG-3' |
|  |  |  |  | R:5'-CCAAGCAAGGGAGATAAGGA-3' |
| TTN-PASS1-2 | *TTN* | SNV | 58 | F:5'-TGCTCAAAGAAGAGCATTCTGA-3' |
|  |  |  |  | R:5'-GGGCTCTTGGGTGATGTTTA-3' |
| TTN-nonPASS1 | *TTN* | SNV | 60 | F:5'-CAGACTGGCACACTTGCATT-3' |
|  |  |  |  | R:5'-CGGAGGTGGGCACTAGATT-3' |
| TTN-nonPASS2 | *TTN* | SNV | 60 | F:5'-CATTGAAAATGCCTCTGGAAA-3' |
|  |  |  |  | R:5'-GAAACTTCTCCCACGCTGTT-3' |
| MUC16-PASS1 | *MUC16* | SNV | 60 | F:5'-CCATGAGAATGCAGGCTTTA-3' |
|  |  |  |  | R:5'-GTGCCTGTCCAGGGTGTAG-3' |
| MUC16-PASS2 | *MUC16* | SNV | 60 | F:5'-CAACATCCTCTCTCTTCACCCTA-3' |
|  |  |  |  | R:5'-AGGTCACAGTTTGGGGCTTA-3' |
| MUC16-nonPASS1 | *MUC16* | SNV | 60 | F:5'-ATAGTCAAGGGACGCATGGA-3' |
|  |  |  |  | R:5'-GCAGACAGGTTGGCAATTCT-3' |
| MUC16-nonPASS2 | *MUC16* | SNV | 58 | F:5'-TTGCATCCATGATTCTGCTC-3' |
|  |  |  |  | R:5'-CCAACCTCAGCCATCACATA-3' |

| Table S4. Description of the filtering statistics and variant counts in five somatic variant callers | | | | | | | | | | | | |
| --- | --- | --- | --- | --- | --- | --- | --- | --- | --- | --- | --- | --- |
|  | Muse | | Mutect2 | | SomaticSniper | | Strelka2 | | VarScan2 | | VarDict | |
| Total variants | 10019 |  | 79933 |  | 9037 |  | 58070 |  | 3656 |  | 37240 |  |
| Filtering statistics |  |  |  |  |  |  |  |  |  |  |  |  |
| PoN | 178 | (1.8%) | 31170 | (39.0%) | 4153 | (46.0%) | 445 | (0.8%) | 727 | (19.9%) | 614 | (1.6%) |
| N_Alt ≥4 | 21 | (0.2%) | 4883 | (6.1%) | 1192 | (13.2%) | 294 | (0.5%) | 168 | (4.6%) | 9 | (0.0%) |
| T_Alt <4 | 675 | (6.7%) | 13538 | (16.9%) | 14 | (0.2%) | 1230 | (2.1%) | 7 | (0.2%) | 12533 | (33.7%) |
| Clustered events | 188 | (1.9%) | 4071 | (5.1%) | 58 | (0.6%) | 576 | (1.0%) | 45 | (1.2%) | 175 | (0.5%) |
| StrandBias | 86 | (0.9%) | 508 | (0.6%) | 2 | (0.0%) | 12 | (0.0%) | 0 | (0.0%) | 11 | (0.0%) |
| Multiallelic site | 4 | (0.0%) | 46 | (0.1%) | 2 | (0.0%) | 8 | (0.0%) | 0 | (0.0%) | 2 | (0.0%) |
| oxodG | 109 | (1.1%) | 1915 | (2.4%) | 0 | (0.0%) | 573 | (1.0%) | 1 | (0.0%) | 382 | (1.0%) |
| Common SNP | 28 | (0.3%) | 111 | (0.1%) | 42 | (0.5%) | 86 | (0.1%) | 25 | (0.7%) | 60 | (0.2%) |
| PASS | 8730 | (87.1%) | 23691 | (29.6%) | 3574 | (39.5%) | 54846 | (94.4%) | 2683 | (73.4%) | 23454 | (63.0%) |
| N_Alt, normal alternative allele in normal samples | | | | | | | | | | | | |
| T_Alt, alternative allele in tumor specimens | | | | | | | | | | | | |

| Table S5. Di-nucleotide mutations are detected by Mutect2, but not in other callers. | | | | | | | | | | | | | | | | | |
| --- | --- | --- | --- | --- | --- | --- | --- | --- | --- | --- | --- | --- | --- | --- | --- | --- | --- |
|  | Mutect2 | |  | Muse | |  | SomaticSniper | |  | Strelka2 | |  | VarScan2 | |  | VarDict | |
| Position | REF | ALT |  | REF | ALT |  | REF | ALT |  | REF | ALT |  | REF | ALT |  | REF | ALT |
| 1:154070268 | AA | CC |  | - | - |  | - | - |  | A | C |  | - | - |  | - | - |
| 1:180916308 | CC | AA |  | C | A |  | - | - |  | C | A |  | - | - |  | - | - |
| 1:220069380 | CA | AT |  | C | A |  | - | - |  | C | A |  | - | - |  | - | - |
| 1:225147097 | TT | AA |  | T | A |  | - | - |  | T | A |  | - | - |  | - | - |
| 1:39683969 | TG | CT |  | T | C |  | T | C |  | T | C |  | T | C |  | - | - |
| 1:960725 | GG | AA |  | - | - |  | - | - |  | G | A |  | - | - |  | - | - |
| 10:73139634 | CC | AT |  | C | A |  | C | A |  | C | A |  | C | A |  | CC | AT |
| 11:17474968 | CA | TG |  | C | T |  | - | - |  | C | T |  | - | - |  | - | - |
| 11:19149265 | CA | GT |  | C | G |  | C | G |  | C | G |  | - | - |  | CA | GT |
| 11:66363264 | CA | AG |  | - | - |  | - | - |  | C | A |  | - | - |  | - | - |
| 11:66369070 | GC | AT |  | G | A |  | G | A |  | G | A |  | G | A |  | GC | AT |
| 12:109945278 | CC | TT |  | - | - |  | - | - |  | C | T |  | - | - |  | - | - |
| 12:119721401 | GG | TT |  | G | T |  | G | T |  | G | T |  | G | T |  | - | - |
| 12:120488652 | AG | CT |  | - | - |  | A | C |  | - | - |  | - | - |  | - | - |
| 12:54343221 | CC | AA |  | C | A |  | - | - |  | C | A |  | - | - |  | - | - |
| 13:29324630 | CA | TC |  | C | T |  | - | - |  | - | - |  | - | - |  | - | - |
| 14:104938281 | GT | TA |  | G | T |  | - | - |  | G | T |  | - | - |  | GT | TA |
| 14:77769497 | GC | AG |  | - | - |  | - | - |  | G | A |  | - | - |  | GC | AG |
| 14:85623229 | AC | TT |  | - | - |  | - | - |  | A | G |  | - | - |  | - | - |
| 15:30925801 | GG | TT |  | G | T |  | G | T |  | G | T |  | G | T |  | GG | TT |
| 15:54014777 | TG | GT |  | T | G |  | T | G |  | T | G |  | T | G |  | TG | GT |
| 15:65400942 | GG | AA |  | G | A |  | - | - |  | G | A |  | - | - |  | GG | AA |
| 15:78533219 | AC | TT |  | A | T |  | A | T |  | A | T |  | A | T |  | AC | TT |
| 16:24789806 | TT | GG |  | T | G |  | - | - |  | - | - |  | - | - |  | T | G |
| 16:68249345 | GC | TG |  | G | T |  | - | - |  | G | T |  | - | - |  | GC | TG |
| 17:10306473 | TC | AA |  | T | A |  | T | A |  | T | A |  | T | A |  | TC | AA |
| 17:17798486 | GG | CA |  | - | - |  | G | C |  | G | C |  | G | C |  | GG | CA |
| 17:9904843 | CC | AA |  | C | A |  | - | - |  | C | A |  | - | - |  | - | - |
| 18:13068391 | CC | TA |  | - | - |  | - | - |  | C | T |  | - | - |  | - | - |
| 18:74576972 | AG | TT |  | A | T |  | A | T |  | A | T |  | A | T |  | AG | TT |
| 19:11905347 | AG | GT |  | A | G |  | - | - |  | A | G |  | - | - |  | AG | GT |
| 19:52706260 | GC | AA |  | G | A |  | - | - |  | G | A |  | - | - |  | - | - |
| 2:214970345 | AC | TT |  | A | T |  | - | - |  | - | - |  | - | - |  | - | - |
| 2:231925692 | CT | AC |  | - | - |  | - | - |  | C | A |  | - | - |  | - | - |
| 2:32250074 | TC | AA |  | - | - |  | - | - |  | T | A |  | - | - |  | - | - |
| 22:36937562 | CC | TT |  | C | T |  | - | - |  | C | T |  | - | - |  | CC | TT |
| 3:172636058 | GG | AA |  | - | - |  | - | - |  | G | A |  | - | - |  | - | - |
| 4:110011305 | CC | TA |  | C | T |  | - | - |  | C | T |  | - | - |  | CC | TA |
| 5:154802122 | AG | GT |  | A | G |  | A | G |  | A | G |  | A | G |  | AG | GT |
| 5:176584479 | GC | TT |  | G | T |  | - | - |  | G | T |  | - | - |  | GC | TT |
| 6:110315355 | GG | TT |  | G | T |  | G | T |  | G | T |  | G | T |  | GG | TT |
| 6:158993922 | CA | AT |  | - | - |  | - | - |  | C | A |  | - | - |  | CA | AT |
| 6:28390595 | CC | AA |  | C | A |  | - | - |  | - | - |  | - | - |  | - | - |
| 7:43444668 | GG | AA |  | G | A |  | G | A |  | G | A |  | G | A |  | GG | AA |
| 7:98223086 | CG | AA |  | - | - |  | - | - |  | C | A |  | - | - |  | - | - |
| 8:119557668 | CC | TT |  | C | T |  | - | - |  | C | T |  | - | - |  | CC | TT |
| 8:142613782 | CG | TA |  | C | T |  | - | - |  | C | T |  | - | - |  | - | - |
| 8:23702726 | GC | CA |  | G | C |  | G | C |  | G | C |  | G | C |  | - | - |
| 9:137278716 | GC | TT |  | G | T |  | G | T |  | G | T |  | G | T |  | GC | TT |
| 9:84298010 | TC | AG |  | T | A |  | T | A |  | T | A |  | - | - |  | TC | AG |
| 9:85962039 | GG | CC |  | G | C |  | - | - |  | G | C |  | - | - |  | GG | CC |
| X:153420705 | GG | TT |  | G | T |  | G | T |  | G | T |  | G | T |  | GG | TT |
| X:48934521 | CA | TT |  | - | - |  | - | - |  | C | T |  | - | - |  | - | - |

| Table S6. The summary of SNVs and indels calls using six variant callers | | | |
| --- | --- | --- | --- |
|  | Unfiltered mutations | Filtered mutations | ≥ 2 callers dataset |
| SNVs |  |  |  |
| Muse | 10,019 (100%) | 8,730 (87.1%) | 8,580 (85.6%) |
| Mutect2 | 70,532 (100%) | 22,432 (31.8%) | 10,218 (14.5%) |
| SomaticSniper | 9,037 (100%) | 3,574 (39.5%) | 3,394 (37.6%) |
| Strelka2 | 58,033 (100%) | 54,823 (94.5%) | 11,305 (19.5%) |
| VarScan2 | 3,430 (100%) | 2,549 (74.3%) | 2,515 (73.3%) |
| VarDict | 36,060 (100%) | 22,674 (62.9%) | 7,285 (20.2%) |
|  |  |  |  |
| Indels |  |  |  |
| Mutect2 | 9,401 (100%) | 1,259 (13.4%) | 253 (2.7%) |
| Strelka2 | 37 (100%) | 23 (62.2%) | 22 (59.5%) |
| VarScan2 | 226 (100%) | 134 (59.3%) | 123 (54.4%) |
| VarDict | 1,180 (100%) | 780 (66.1%) | 204 (17.3%) |
| ≥ 2, mutations called by at least two callers  The percentage formula: group mutations x 100/unfiltered mutations | | | |

| Table S7. List of most frequency False-positive genes | | | | | | |
| --- | --- | --- | --- | --- | --- | --- |
| Genes | Total mutations | IGV PASS | |  | IGV non-PASS | |
| *MUC16* | 2,121 | 21 | (1.0%) |  | 2,100 | (99.0%) |
| *MUC19* | 1,906 | 6 | (0.3%) |  | 1,900 | (99.7%) |
| *KMT2D* | 108 | 36 | (33.3%) |  | 72 | (66.7%) |
| *TTN* | 136 | 69 | (50.7%) |  | 67 | (49.3%) |
| *HERC2* | 64 | 23 | (35.9%) |  | 41 | (64.1%) |
| *LRP1B* | 65 | 27 | (41.5%) |  | 38 | (58.5%) |
| *NEB* | 70 | 34 | (48.6%) |  | 36 | (51.4%) |
| *SKOR2* | 45 | 15 | (33.3%) |  | 30 | (66.7%) |
| *ZFHX4* | 53 | 27 | (50.9%) |  | 26 | (49.1%) |
| *DNAH1* | 31 | 6 | (19.4%) |  | 25 | (80.6%) |
| *PLEC* | 53 | 29 | (54.7%) |  | 24 | (45.3%) |
| *ABCA13* | 49 | 26 | (53.1%) |  | 23 | (46.9%) |
| *RYR2* | 52 | 33 | (63.5%) |  | 19 | (36.5%) |
| *EPPK1* | 39 | 21 | (53.8%) |  | 18 | (46.2%) |
| *FMN2* | 34 | 15 | (44.1%) |  | 19 | (55.9%) |
| *FAT2* | 41 | 24 | (58.5%) |  | 17 | (41.5%) |
| *FAT3* | 40 | 23 | (57.5%) |  | 17 | (42.5%) |
| *HYDIN* | 29 | 12 | (41.4%) |  | 17 | (58.6%) |
| *CUBN* | 45 | 29 | (64.4%) |  | 16 | (35.6%) |
| *NOTCH1* | 37 | 22 | (59.5%) |  | 15 | (40.5%) |

| Table S8. Confirmation of mutations by Sanger sequencing in the top 4 IGV non-PASS genes | | | | | | | |
| --- | --- | --- | --- | --- | --- | --- | --- |
|  |  | IGV PASS | |  | IGV non-PASS | |  |
|  |  | Sanger confirmation | |  | Sanger confirmation | |  |
| Genes | Mutations | WT | Mutant |  | WT | Mutant |  |
| *MUC19* | 8 | 0 (0%) | 2 (100%) |  | 6 (100%) | 0 (0%) |  |
| *MUC16* | 18 | 0 (0%) | 2 (100%) |  | 16 (100%) | 0 (0%) |  |
| *TTN* | 4 | 0 (0%) | 2 (100%) |  | 2 (100%) | 0 (0%) |  |
| *KMT2D* | 4 | 0 (0%) | 2 (100%) |  | 2 (100%) | 0 (0%) |  |

| Table S9. Most frequencies mutated genes and mutations validated by IGV | | | | | | | | | | |  |
| --- | --- | --- | --- | --- | --- | --- | --- | --- | --- | --- | --- |
|  | Unfiltered mutations (n=1324) | | | |  | Filtered mutations (n=954) | | | |  | |
|  | IGV PASS | | IGV non-PASS | |  | IGV PASS | | IGV non-PASS | | *P*-value | |
| Total | 741 | (56.0%) | 583 | (44.0%) |  | 706 | (74.0%) | 248 | (26.0%) | <0.001* | |
| Variant callers |  |  |  |  |  |  |  |  |  |  | |
| Muse | 226 | (84.3%) | 42 | (15.7%) |  | 223 | (86.8%) | 34 | (13.2%) | 0.632 | |
| Mutect2 | 339 | (42.7%) | 454 | (57.3%) |  | 306 | (64.7%) | 167 | (35.3%) | <0.001* | |
| SomaticSniper | 144 | (84.7%) | 26 | (15.3%) |  | 141 | (97.2%) | 4 | (2.8%) | <0.001* | |
| Strelka2 | 642 | (85.0%) | 113 | (15.0%) |  | 636 | (87.6%) | 90 | (12.4%) | 0.151 | |
| VarScan2 | 124 | (90.5%) | 13 | (9.5%) |  | 121 | (96.0%) | 5 | (4.0%) | 0.077 | |
| VarDict | 217 | (94.3%) | 13 | (2.7%) |  | 211 | (95.5%) | 10 | (4.5%) | 0.586 | |
|  |  |  |  |  |  |  |  |  |  |  | |
| Mutation calling times | |  |  |  |  |  |  |  |  |  | |
| ≥ 2 callers | 325 | (86.4%) | 51 | (13.6%) |  | 313 | (88.7%) | 40 | (11.3%) | 0.362 | |
| ≥ 3 callers | 246 | (94.6%) | 14 | (5.4%) |  | 242 | (96.0%) | 10 | (4.0%) | 0.448 | |
| ≥ 4 callers | 187 | (95.9%) | 8 | (4.1%) |  | 184 | (96.3%) | 7 | (3.7%) | 0.824 | |
| ≥ 5 callers | 126 | (96.9%) | 4 | (3.1%) |  | 125 | (96.9%) | 4 | (3.1%) | 0.991 | |
| 6 callers | 68 | (98.6%) | 1 | (1.4%) |  | 68 | (98.6%) | 1 | (1.4%) | 1.000 | |
| *P* value was calculated by chi-square | | | | | | | | | | |  |
| *, *P* < 0.05 | | | | | | | | | | |  |

| Table S10. Most frequently mutated genes in OSCC (n=50) | | | | | | |
| --- | --- | --- | --- | --- | --- | --- |
| Genes | Driver gene in database | Our study (n=50) | |  | TCGA (n=507) | |
|  |  | Patients with mutation | Mutation frequency |  | Patients with mutation | Mutation frequency |
| *SKOR2* | N | 7 | 0.14 |  | NA | NA |
| *CPLANE1* | N | 6 | 0.12 |  | NA | NA |
| *CCDC168* | N | 6 | 0.12 |  | NA | NA |
| *STARD9* | N | 6 | 0.12 |  | NA | NA |
| *NSD2* | N | 5 | 0.10 |  | NA | NA |

| Table S11. Most frequently mutated genes in OSCC (n=50) | | | | | | |  |
| --- | --- | --- | --- | --- | --- | --- | --- |
| Genes | Driver gene in database | Our study (n=50) | |  | TCGA-OSCC (n=387) | | |
|  |  | Patients with mutation | Mutation frequency |  | Patients with mutation | Mutation frequency | |
| *TP53* | Y | 31 | 0.62 |  | 264 | 0.68 | |
| *FAT1* | Y | 20 | 0.40 |  | 99 | 0.26 | |
| *NOTCH1* | Y | 14 | 0.28 |  | 75 | 0.19 | |
| *TTN* | N | 13 | 0.26 |  | 163 | 0.42 | |
| *CASP8* | Y | 11 | 0.22 |  | 51 | 0.13 | |
| *RYR2* | N | 10 | 0.20 |  | 35 | 0.09 | |
| *FMN2* | N | 9 | 0.18 |  | 25 | 0.06 | |
| *KMT2D* | Y | 9 | 0.18 |  | 49 | 0.13 | |
| *ABCA13* | N | 8 | 0.16 |  | 27 | 0.07 | |
| *EPHA2* | Y | 8 | 0.16 |  | 24 | 0.06 | |
| *FAT2* | N | 8 | 0.16 |  | 30 | 0.08 | |
| *FAT3* | Y | 8 | 0.16 |  | 31 | 0.08 | |
| *HYDIN* | N | 8 | 0.16 |  | 17 | 0.04 | |
| *LRP1B* | Y | 8 | 0.16 |  | 61 | 0.16 | |
| *NEB* | N | 8 | 0.16 |  | 31 | 0.08 | |
| *PLEC* | N | 8 | 0.16 |  | 51 | 0.13 | |
| *ZFHX4* | N | 8 | 0.16 |  | 30 | 0.08 | |
| *ALPK3* | N | 7 | 0.14 |  | 16 | 0.04 | |
| *ASXL1* | Y | 7 | 0.14 |  | 11 | 0.03 | |
| *CSMD3* | N | 7 | 0.14 |  | 53 | 0.14 | |
| *CUBN* | N | 7 | 0.14 |  | 29 | 0.07 | |
| *EPPK1* | N | 7 | 0.14 |  | 22 | 0.06 | |
| *HERC2* | N | 7 | 0.14 |  | 32 | 0.08 | |
| *PIK3CA* | Y | 7 | 0.14 |  | 69 | 0.18 | |
| *PKD1L1* | N | 7 | 0.14 |  | 13 | 0.03 | |
| *PTPRT* | Y | 7 | 0.14 |  | 16 | 0.04 | |
| *SKOR2* | N | 7 | 0.14 |  | #N/A | #N/A | |
| *SORCS3* | N | 7 | 0.14 |  | 20 | 0.05 | |
| *USP9X* | Y | 7 | 0.14 |  | 17 | 0.04 | |
| *WDFY3* | N | 7 | 0.14 |  | 14 | 0.01 | |
|  |  |  |  |  |  |  | |
|  | | | | | | | |

| Table S12. The relationship of clinical parameters and mutated genes | | | | | |
| --- | --- | --- | --- | --- | --- |
| Variables |  | No. of patient with Mutant | No. of patient with WT |  | *P*-value^a^ |
| *ASXL1* |  |  |  |  |  |
| Age | <59.6 | 0 | 24 |  | 0.010* |
|  | ≥59.6 | 7 | 19 |  |  |
|  |  |  |  |  |  |
| *FAT1* |  |  |  |  |  |
| Clinical stage | I-III | 1 | 10 |  | 0.033* |
|  | IV | 19 | 20 |  |  |
| T stage | T1-T3 | 2 | 11 |  | 0.050 |
|  | T4 | 18 | 19 |  |  |
|  |  |  |  |  |  |
| *PKD1L1* |  |  |  |  |  |
| Histological grade | Well | 2 | 34 |  | 0.044* |
|  | Moderate-Poor | 4 | 10 |  |  |
|  |  |  |  |  |  |
| *TTN* |  |  |  |  |  |
| Perineural invasion | No | 12 | 21 |  | 0.038* |
|  | Yes | 1 | 16 |  |  |
|  |  |  |  |  |  |
| *FAT3* |  |  |  |  |  |
| Histological grade | Well | 8 | 28 |  | 0.087 |
|  | Moderate-Poor | 0 | 14 |  |  |
|  |  |  |  |  |  |
| *FMN2* |  |  |  |  |  |
| T stage | T1-T3 | 0 | 13 |  | 0.093 |
|  | T4 | 8 | 29 |  |  |
| Histological grade | Well | 8 | 28 |  | 0.087 |
|  | Moderate-Poor | 0 | 14 |  |  |
| ^a^*P*-value were determined with Fisher's exact test | | | | | |
| **P* < 0.05 | | | | | |
